# Beat Pilot Tone (BPT): Simultaneous MRI and RF motion sensing at arbitrary frequencies

**DOI:** 10.1002/mrm.30150

**Published:** 2024-06-14

**Authors:** Suma Anand, Michael Lustig

**Affiliations:** Electrical Engineering and Computer Sciences, University of California, Berkeley, California

**Keywords:** microwave, motion sensing, pilot tone, radiofrequency

## Abstract

**Purpose:**

To introduce a simple system exploitation with the potential to turn MRI scanners into general-purpose radiofrequency (RF) motion monitoring systems.

**Methods:**

Inspired by Pilot Tone (PT), this work proposes Beat Pilot Tone (BPT), in which two or more RF tones at arbitrary frequencies are transmitted continuously during the scan. These tones create motion-modulated standing wave patterns that are sensed by the receiver coil array, incidentally mixed by intermodulation in the receiver chain, and digitized simultaneously with the MRI data. BPT can operate at almost any frequency as long as the intermodulation products lie within the bandwidth of the receivers. BPT’s mechanism is explained in electromagnetic simulations and validated experimentally.

**Results:**

Phantom and volunteer experiments over a range of transmit frequencies suggest that BPT may offer frequency-dependent sensitivity to motion. Using a semi-flexible anterior receiver array, BPT appears to sense cardiac-induced body vibrations at microwave frequencies (≥1.2 GHz). At lower frequencies, it exhibits a similar cardiac signal shape to PT, likely due to blood volume changes. Other volunteer experiments with respiratory, bulk, and head motion show that BPT can achieve greater sensitivity to motion than PT and greater separability between motion types. Basic multiple-input multiple-output (4 × 22 MIMO) operation with simultaneous PT and BPT in head motion is demonstrated using two transmit antennas and a 22-channel head-neck coil.

**Conclusion:**

BPT may offer a rich source of motion information that is frequency-dependent, simultaneous, and complementary to PT and the MRI exam.

## 1 | INTRODUCTION

Motion is the most common unanticipated event in a clinical MRI examination.^1^ Even when anticipated, such as with respiratory and cardiac motion, motion during the examination degrades image quality, resulting in repeated exams and increased costs.^2^ Moreover, as spatial resolution continues to improve, motion is accentuated and causes greater image corruption.^3–5^

Simple motion mitigation techniques, like breath-holding, may prove unreliable and limit scan options. For instance, three-dimensional imaging requires longer scan-time than a single breath-hold duration. Instead, modern motion correction techniques require measuring the motion during the exam and correcting artifacts prospectively, retrospectively, or both. While it is possible to use only the MR data itself for correction,^6–8^ external motion monitoring signals or images offer a rich source of information that may improve the quality and speed of the correction.^9–16^

Existing motion sensing systems in MRI may have limitations in sensitivity, ease of general use, or patient comfort. Cameras^17,18^ are accurate but require a line-of-sight path that is often blocked by the MRI coils and are limited in penetration depth.^9^ NMR- and MRI-based navigators^4–6,12,13,16^ require sequence modifications and are adopted in limited applications.^19^ Radiofrequency (RF) sensing^20–28^ can detect a variety of motion types with high sensitivity and without contact; however, existing solutions are lacking in either sensitivity or generality. “RF coil” (RFC) sensors^20,22–24^ use the MRI receiver or transmitter coils as motion sensors but are tied to the Larmor frequency, thus limiting their inherent sensitivity, while ultra-high frequency RF sensors such as radars^26–28^ require significant engineering efforts to be integrated with the MRI scanner, hindering general implementation.

**FIGURE 1:**
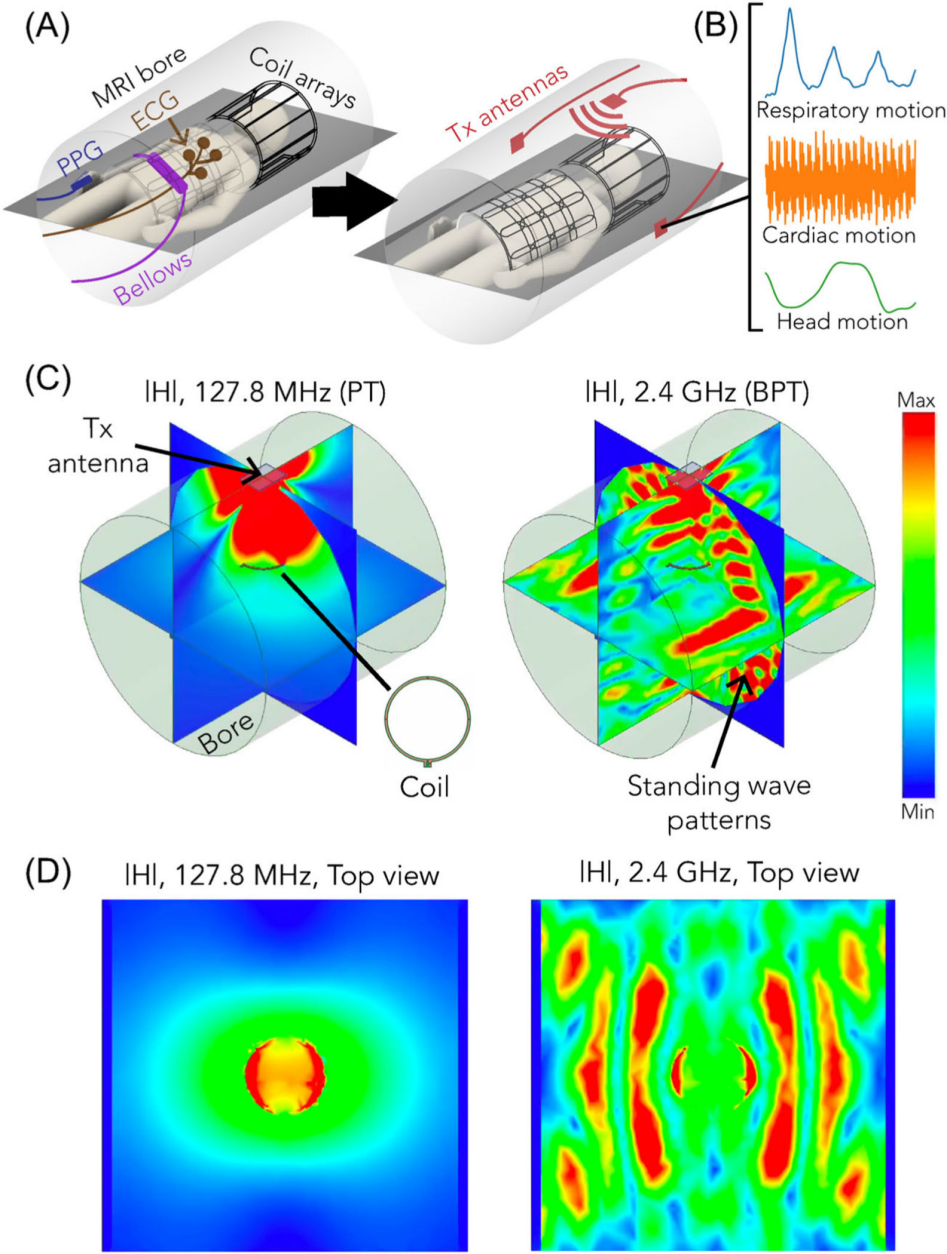
Beat Pilot Tone (BPT) concept. (A) Conventional MRI suite sensors (left) are bulky and require contact with the patient, while our method (right) utilizes MRI coil arrays and requires a transmit (tx) antenna in or near the bore to provide motion estimates (B). Multiple antennas can be used for multiple input multiple output (MIMO) operation. (C) Simulated magnitudes of the H-field generated by a tx dipole antenna at 127.8 MHz (left) and 2.4 GHz (right), windowed to the same relative levels. A receiver coil tuned to 127.7 MHz is placed 10 cm above the center of the bore. At 2.4 GHz, standing wave patterns emerge. (D) Top view of |H| at (left) 127.8 MHz and (right) 2.4 GHz in the plane of the coil.

In this paper, we introduce Beat Pilot Tone (BPT), a simple system exploitation with the potential to turn any MRI scanner into a general-purpose RF motion monitoring system. BPT involves transmitting RF tones that interact with the body and MRI bore to create motion-modulated standing wave patterns (Figure 1C,D). These waves are sensed by the receiver coil array, incidentally mixed through nonlinear intermodulation in the receiver chain, and digitized simultaneously with the MRI data. BPT can therefore operate at almost any frequency, as long as the intermodulation lies within the bandwidth (BW) of the receivers. To distinguish between the transmitted and received BPT, we denote the entire concept as “BPT,” the transmit BPT field as “BPT-Tx,” and the sensed BPT signals after mixing as “BPT-Rx.” Figure 1C,D shows a simulated BPT-Tx at 2.4 GHz for a transmitter placed at the top of the bore and a receiver coil 10 cm above the center.

Expanding the transmit frequency range offers the potential for frequency-dependent motion sensitivity; however, understanding its mechanism requires modeling electromagnetic (EM) wave effects at higher frequencies. We model these effects in simulation and validate them in experiment over a range of transmit frequencies (127.8 MHz–2.5278 GHz). We show that the standing wave patterns from BPT-Tx increase in complexity and percent modulation with frequency. We evaluate the frequency-dependent motion sensitivity of BPT-Rx in phantom and volunteer experiments for common motion types (respiratory, bulk, cardiac, and head motion) using different coil arrays. BPT-Rx at microwave frequencies has the potential to separate motion types more easily than a well-established RFC method known as Pilot Tone (PT), which uses a single RF tone at ∼127.8 MHz on our 3T MRI scanner. Moreover, BPT-Rx cardiac signal on volunteers reflects blood volume changes below 1.2 GHz and correlates strongly with small body vibrations (displacement ballistocardiogram; dBCG) at higher frequencies using a semi-rigid array. Finally, we show the potential for quantitative motion correction when using BPT as a frequency-multiplexed multiple-input multiple-output (MIMO) system. With two transmitters placed at different locations, BPT-Rx shows differences between two different head motions (nodding “yes” and shaking “no”) in a pattern similar to motion derived from image registration. This approach has been demonstrated for retrospective head motion correction in preliminary experiments.^29^ BPT thus offers the potential for simultaneous MR imaging and motion monitoring at arbitrary frequencies in many applications.

## 2 | METHODS

We describe the general method of BPT (Section 2.1) and its hardware implementation (Section 2.2). We then hypothesize where and how BPT-Rx is created (Section 2.3). We examine the mechanism of BPT in EM simulations (Section 2.4) and experiments (Section 2.5) which show its sensitivity to small vibrations.

### 2.1 | BPT signal reception

BPT builds off of PT^24,25^; however, BPT is additionally sensitive to EM wave effects, potentially allowing detection of subtler movements. RFC methods share a similar mechanism for sensing motion. Motion changes the load impedance to the transmitter or receiver coils, resulting in reflected power or changes in magnitude and phase.^20,23,32^ Load impedance is dominated by the impedance of the body at clinical field strengths.^20^ Additionally, motion can change tissue conductivity^20^ and distance between the coil and the body.^33^ RFC methods measure changes in load impedance passively or actively. A passive method known as a “noise navigator” tracks changes in the variance of received noise.^22^ Active methods measure reflected power at the transmitter^20,23^ or voltage changes at the receiver coils due to the superposition of transmitted and induced EM fields (PT).^32^ In PT, the transmitter and receiver coils are distinct; thus, variations in distance between them can lead to additional magnitude and phase changes.^34^

In PT, a tone near the Larmor frequency is transmitted continuously and detected by the receiver coils. For some motion types, the transmitter and/or receiver coils must be close to the subject for high signal-to-noise ratio (SNR) sensing, such as cardiac motion.^34^ Due to its ability to sense many motion types with minimal hardware additions, PT has been integrated into commercial systems, offering valuable respiratory, cardiac, and head motion information.^32,35–38^ However, RFC methods are fundamentally limited in motion sensitivity, as measured by percent modulation: some studies suggest between 5% and 20% modulation for breathing^20,33^ and 1%–2% for cardiac motion.^20,34^ Moreover, these methods may not translate well to low-field scanners. Because body impedance is weaker at lower frequencies, RFC methods may suffer from lower SNR in low-field systems.^20^

**FIGURE 2:**
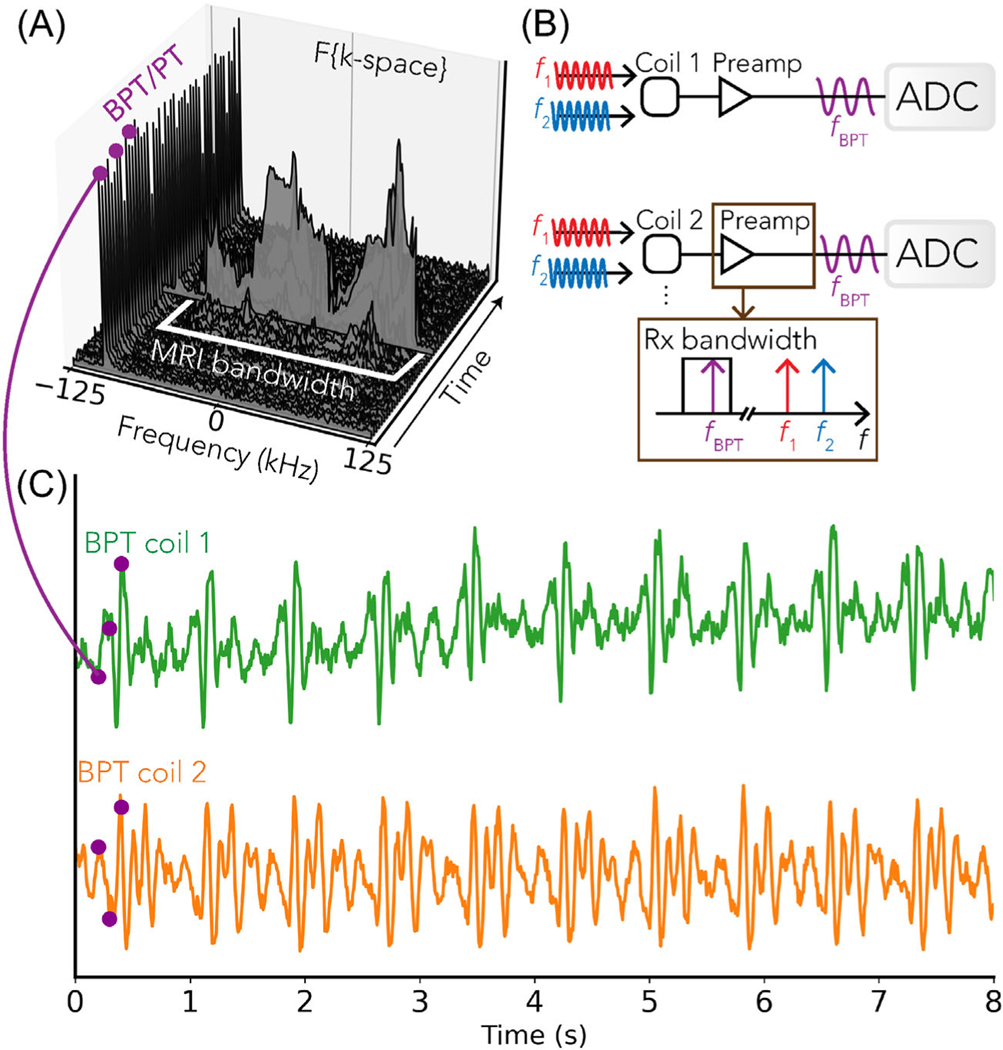
Pipeline for BPT extraction. (A) BPT-Rx appears as a peak in the Fourier transform of each k-space line. The intermodulation frequency $f_{\text{BPT}}$ falls within the bandwidth (BW) of the receiver but outside the BW of the MR image data. (B) Model of the receiver chain: the electromagnetic fields at frequencies $f_{1}$ (red) and $f_{2}$ (blue) are sensed by MRI receiver coils and mixed by the preamplifiers to generate an intermodulation product at frequency $f_{\text{BPT}}$.^30,31^ (C) Example of the magnitude of the BPT-Rx signal from two receiver coils, corresponding to the time-frequency plot in (A) and showing amplitude modulation due to cardiac motion in a breath-holding volunteer.

In contrast, BPT is not tied to the Larmor frequency. Rather than transmitting a single RF tone, BPT uses two tones and exploits a nonlinear property of the receiver chain known as *intermodulation* for reception. BPT can be separated into two pieces: the transmit field (BPT-Tx) and the received signal after mixing (BPT-Rx). Two tones with frequencies that may lie far outside the MR BW are transmitted into the bore, creating two BPT-Tx fields. The BPT-Tx fields interact with the bore and body, are sensed by the receiver coil elements, and serendipitously mixed by intermodulation, creating a new signal called an intermodulation product or distortion (IMD) that we denote as BPT-Rx. Intermodulation is a property of nonlinear devices (e.g., diodes, transistors, etc.) in which inputs at frequencies $f_{\text{1}}$ and $f_{\text{2}}$ can create an output at a new frequency $f_{\text{BPT}}$,^39^ where:

(1) $$f_{\text{BPT}}=mf_{2}+nf_{1},$$

and m and n are signed integers. $f_{\text{1}}$ and $f_{\text{2}}$ are chosen such that the IMD at $f_{\text{BPT}}$ will fall close to the Larmor frequency. For example, two tones transmitted at $f_{1}=2.4\;\text{GHz}$, $f_{2}=2.5278\;\text{GHz}$ will have an IMD at frequency

(2) $$f_{\text{BPT}}=f_{2}-f_{1}=127.8\;\text{MHz},$$

which is within our GE MR750W (GE Healthcare) 3T scanner BW. This example uses two tones to create two BPT-Tx fields, resulting in a single BPT-Rx via second-order intermodulation. Both BPT-Rx and PT appear as a peak in the Fourier transform of each k-space line that is separable from the MR data (Figure 2A).

### 2.2 | Hardware implementation

**FIGURE 3:**
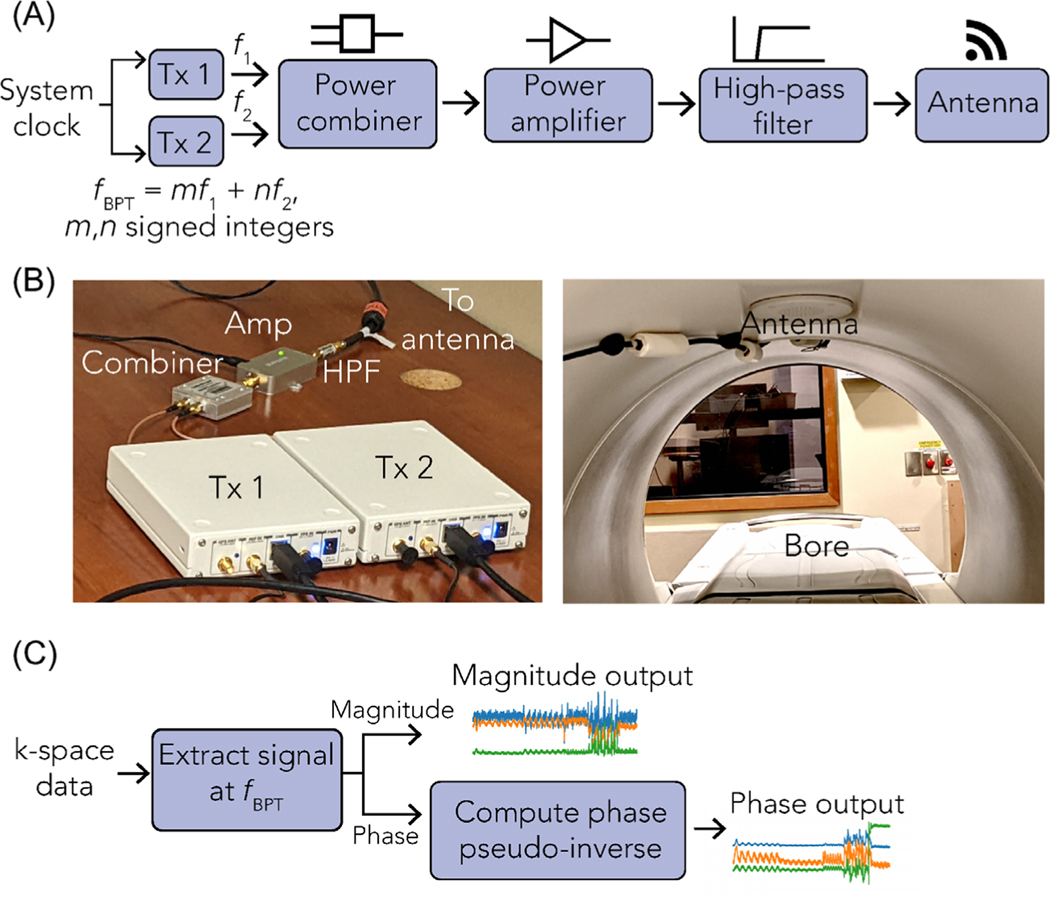
Acquisition and processing pipeline. (A) The acquisition pipeline, in which two tones were generated with two transmitters (e.g., software-defined radios [SDRs]), combined, amplified, high-pass filtered, and transmitted with an antenna. (B) Photo of a sample setup, in which the transmitters (left) are USRP B200 SDRs and an antenna (right) is placed at the top of the bore. (C) Basic reconstruction pipeline, where the complex signal at $f_{\text{BPT}}$ is extracted from the raw k-space data. The phase is computed by taking the pseudo-inverse of all possible reference coils relative to a chosen reference coil (Data S1).

BPT-Tx can be implemented with off-the-shelf and low-cost hardware (Figure 3). We used two software-defined radios (SDRs) to produce the two tones (Ettus Research B200; National Instruments), synchronized to the system 10 MHz clock. The tones were combined, amplified, filtered, and transmitted using an antenna placed inside or outside the bore. The filter removed intermodulation from the transmit amplifier in the transmitted tones. The specific hardware components are listed in Tables S1 and S2.

### 2.3 | Intermodulation properties

Intermodulation may occur by any non-linear component in the receiver chain. This could be passive, like diodes used for coil detuning and protection, or active, like the preamplifier circuits (preamps) themselves. We hypothesize that the preamps are the likely culprit because little BPT-Tx transmit power (<20 dBm at the transmit antenna) is sufficient to induce IMD at a similar amplitude to the MR signal. We measured the strength of the IMD on a representative MR preamplifier (Clinical MR Solutions, LLC; Figure S1). Using a spectrum analyzer (FieldFox N9918A; Keysight Technologies), the input power was swept from −14 dBm to +2 dBm, and the power of the IMD was measured, along with the output power at 2.4 and 2.5278 GHz (“fundamental”). Since the MR signal power ranges from −75 to −40 dBm at 3T,^40^ Figure S1 shows that it is possible to obtain an IMD power close to that of the MR signal (>−70 dBm) with little BPT-Tx power (−10 dBm), suggesting that intermodulation in the preamp is the likely mechanism of BPT-Rx.

### 2.4 | EM coil motion simulations

BPT-Rx, like PT and other RFC methods, senses changes in the EM field. However, when using high frequencies (∼2.4 GHz), it is additionally sensitive to EM wave effects. We simulated these effects in a basic simulation where BPT-Rx was computed across a range of frequencies in an ideal vacuum-filled bore, with three virtual coils moving along the z-axis. We validated the accuracy of the simulation in an experiment. Though the simulation and experiment do not capture the complexity of BPT on a human subject, they show the key differences between BPT and RFC methods: namely, that standing wave patterns are produced by the boundary conditions of the setup, these waves are sampled and mixed to create BPT-Rx, and that this mechanism may enable tunable sensitivity to motion depending on the transmit frequencies.

**FIGURE 4:**
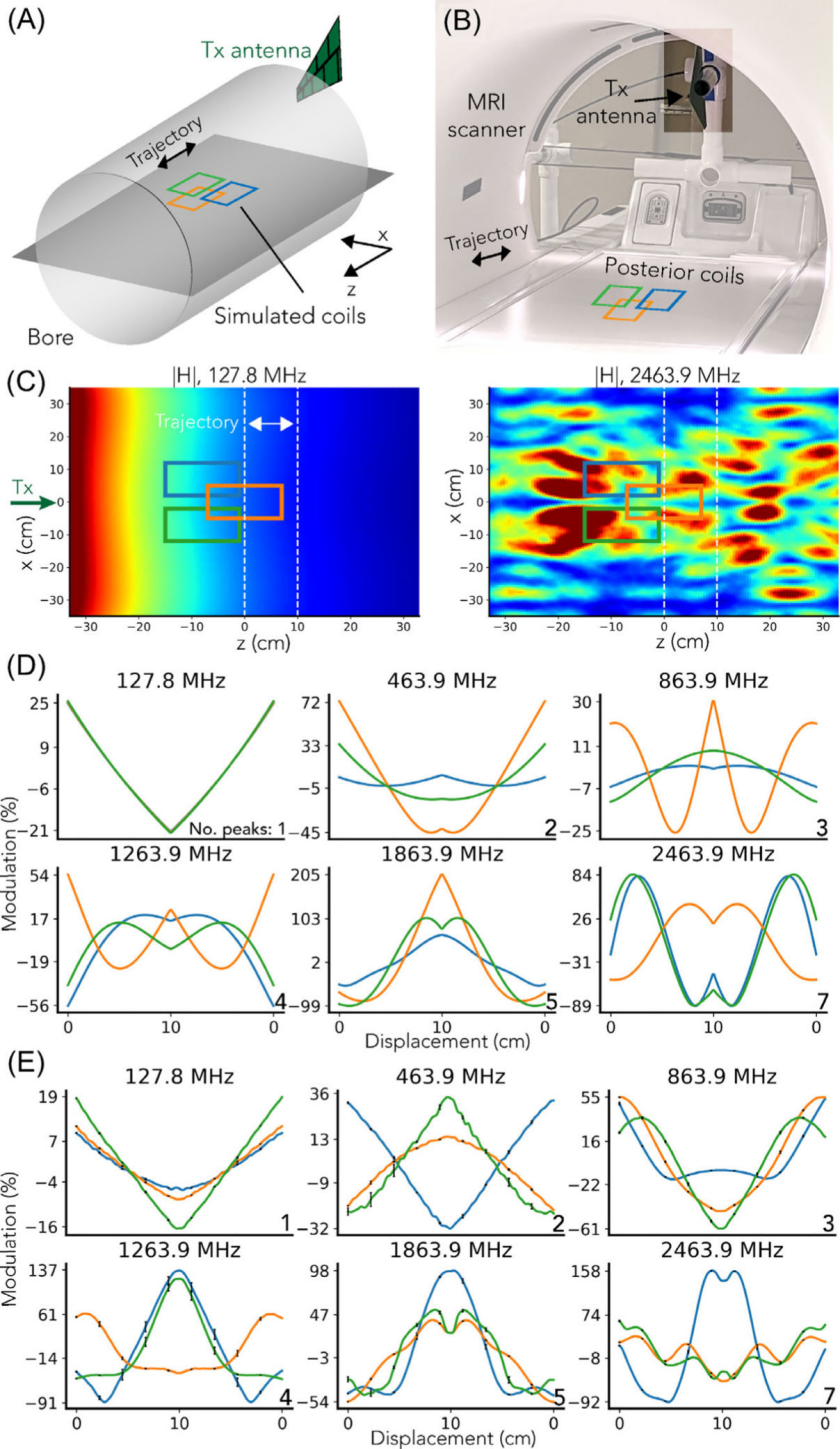
BPT finite-element electromagnetic (EM) field simulations. (A) Simulation setup for an idealized bore. Flux was computed through rectangular coils moving along the z-axis. (B) The experimental setup. (C) |H| at the central slice of the bore at (left) 127.8 MHz and (right) 2463.9 MHz (product of |H| at 2400 and 2527.8 MHz). Coil locations are shown by the colored rectangles, range of motion by white dashed lines, and tx antenna location by the green arrow. While |H| at 127.8 MHz decays over distance, |H| at 2463.9 MHz shows standing wave patterns. (D) Simulated magnitude of BPT-Rx in percent modulation units, with the maximum theoretical number of peaks on the bottom right. (E) The measured magnitude of BPT-Rx for three coils in the posterior array. The signals were averaged over five periods, with SD in black. The number of peaks and level of modulation increase with frequency, showing the same trends in simulation and experiment.

The simulations were conducted with the finite element solver High-Frequency Structure Simulator (Ansys). The field was transmitted by an antenna outside the bore (Figure 4A) and sensed by three virtual coils. The coils were moved 10 cm back and forth along the z-direction (Figure 4A). The bore was simulated as a perfect electrical conductor and sized to match the GE 3T MR750W system used for all experiments (70 cm in diameter, 135 cm in length). Its calculated cutoff frequency is 251.1 MHz.^41^ The simulated antenna was a wideband log periodic antenna (600 MHz–6 GHz) modeled after the antenna used in experiments (Agatige). The EM field was simulated as a multifrequency simulation with frequencies 127.8, 400, 527.8, 800, 927.8, 1200, 1327.8, 1800, 1927.8, 2400, and 2527.8 MHz.

By the Maxwell–Faraday law of induction, the received voltage is proportional to the flux $\text{Φ}=\int \vec{H}\cdot \vec{d}A$. Consequently, PT was simulated by computing Φ at 127.8 MHz, and BPT-Rx by computing the product of Φ through the three coils for each BPT-Tx frequency pair. The coils were modeled as rectangular surfaces that were matched in size (10 × 14 cm^2^) and position to the posterior coils used in experiments (Figure 4B). The magnitude of Φ was converted to percent modulation by dividing by the mean, i.e. for a signal x of length N,

(3) $$\%\text{mod}\;=\left(\frac{\text{x}}{\frac{1}{N}\sum \text{x}}-1\right)\times 100.$$

In our experimental validation, we measured PT and BPT-Rx at the same frequencies and displacements as the simulated values. We used the hardware setup in Figure 3A to transmit BPT-Tx with a wideband amplifier (ZX60–83MP-S+; Minicircuits), and a ∼50dB Larmor frequency band-stop filter (ZX75BS-125-S+; Minicircuits). The BPT-Tx powers ranged from 5 to 10 dBm at the antenna in order to result in the same received level (explained in Section 2.6). To transmit PT, we used a waveform generator (SDG6022X; Siglent Technologies) and a power of −28 dBm. We inductively suppressed common-mode parasitics from the transmit cable using air-core winding of the coaxial transmission line. We moved a posterior receiver array back and forth along the z-axis using Rocker, an application that moves the scanner bed bidirectionally using the built-in motor (Figure 4B). To ensure repeatability, we repeated the motion for five periods, aligned the signals, and plotted the mean signal, with black error bars indicating SD. Magnitude BPT signals were extracted, low-pass filtered with zero group delay and a cutoff of 2 Hz, then converted to percent modulation.

Figure 4C,D shows the results of the simulation. The main differences between BPT and PT are due to the nonlinear reception of BPT-Rx and the frequency-dependent characteristics of the MRI bore, which acts as a cylindrical waveguide.^42^ The simulated |H| at 127.8 MHz (Figure 4C, left) decays with distance from the transmitter because it is below the waveguide cutoff frequency.^42^ However, at frequencies greater than the cutoff, such as 2.4 GHz, the transmitted tone produces a standing wave (BPT-Tx) that forms spatially-varying patterns (Figure 4C, right).^42^

After evaluating the simulation qualitatively and quantitatively, we found that key features of the simulation are validated by experiment. In simulation and experiment, the number of peaks, level of modulation, and spatial variation in PT and BPT-Rx signals increase with frequency (Figure 4D,E). The first subplot is PT at 127.8 MHz; the remaining plots are BPT-Rx, labeled by the average of the two transmit frequencies. The simulated PT transmit field decays in space and has no nonlinear sensing; consequently, there is a single peak in the received waveform, and adjacent coil signals appear similar in shape and level of modulation. This also holds true in the measured PT (Figure 4E). However, in BPT-Rx, interaction with the bore and nonlinear sensing cause the predicted number of peaks to increase over frequency (explained in the Appendix S1). The maximum peak count (bottom right of each subplot in Figure 4D,E) is consistent with the simulated and experimental data. Moreover, because each coil samples the local field, adjacent coil signals appear to have different shapes and levels of modulation from one another. Finally, there is an increase in the level of modulation over frequency. The simulated modulation ranges from −21 to 25% at 127.8 MHz and −89 to 84% at 2463.9 MHz. This is a 3.8× increase in sensitivity. While the measured data shows a similar modulation range for PT, the measured BPT-Rx range at 2463.9 MHz appears larger than the simulated range. Discrepancies are discussed in Section 4.

### 2.5 | Coil vibration experiment

We performed a phantom experiment to evaluate the sensitivity of BPT-Rx to small motions by measuring vibrations of the receiver coil across a range of BPT-Tx transmit frequencies. Using the same transmit antenna and a combined anterior/posterior semi-flexible array (“GEM” anterior array; GE Healthcare), we caused the coils to vibrate by placing the array on a plastic structure and moving it abruptly. The scanner cradle was moved at the maximum speed (100 mm/s) and a small displacement (1 cm), then stopped for 5 s for vibrations to decay. PT and BPT-Rx were measured sequentially and continuously during both cradle motion and rest periods. The data was low-pass filtered with zero group delay and cutoffs of 1 Hz for frequencies <863.9 MHz and 5 Hz for frequencies ≥863.9 MHz for better visualization.

We validated the origin of these fluctuations by comparing BPT-Rx at the two highest transmit frequencies to displacement calculated from accelerometer measurements. A tri-axial SCL3300 accelerometer (Murata Electronics) was controlled by an Arduino Pro Mini (Arduino) and synchronized to BPT and PT via a Transistor-Transistor Logic signal from the scanner. The displacement was computed from acceleration by high-pass filtering with a cutoff of 2.5 Hz, then integrating the signal twice. BPT-Rx data were low-pass filtered with a cutoff of 5 Hz. Accelerometer and BPT-Rx measurements were obtained sequentially in order to prevent accelerometer noise from corrupting the BPT-Rx acquisition. RF excitation and gradients were turned off.

### 2.6 | Volunteer experiments

To explore BPT’s motion sensitivity in volunteers, we performed volunteer experiments with respiratory, bulk, cardiac, and head motion. The coil arrays used in these experiments varied in rigidity and level of contact with the subject. While the coils in the respiratory, bulk, and cardiac experiments touched and moved with the subject’s body, the coils in the head motion experiment were fixed and not in close contact with the subject, potentially leading to different mechanisms of motion sensing. The BPT transmit antenna(s) were placed away from the subject in most experiments, while many of the sensors used for comparison (PT antenna, PPG, ECG, and accelerometer) were placed on the subject’s chest or abdomen. All scans were acquired on healthy volunteers after obtaining IRB approval and informed consent. Floating cable traps were placed on the cable connected to the transmit antenna(s) to suppress common-mode currents induced by the MRI transmit RF field.^43^ The received levels of BPT-Rx and PT were equalized by adjusting the transmit powers until the root-sum-square of the amplitude of BPT-Rx and PT peaks across coils were approximately equal. The received levels for different BPT-Tx transmit frequencies were equalized in the same way, resulting in a range of transmit powers. All filtering had zero group delay.

The maximum BPT-Tx power used (100 mW) would produce energy absorption of 0.08 mW/cm^2^ in the worst case, which is well below the limit of 5 mW/cm^2^ given by the Federal Communications Commission.^44^ We estimated this by assuming isotropic radiation from the antenna at a distance of 10 cm from the subject, as in the head motion experiment. The power density $P_{d}$ is then 100 mW divided by the surface area of a sphere with radius r=10cm:

(4) $$P_{d}=100/\left(4\pi r^{2}\right)=0.08\text{mW}/{\text{cm}}^{2}.$$

#### 2.6.1 | Respiratory motion experiment

A volunteer was asked to perform different breathing types: chest breathing, stomach breathing, rapid-shallow breathing, and breathing with simultaneous bulk motion of the chest (Figure 6). The bulk motion consisted of rotation of within ±3 degrees about the head-foot axis and translation between −15 and 5 mm in the left-right and anterior–posterior axes (Figure S2). PT and BPT-Tx were transmitted simultaneously at frequencies of 127.8 MHz and 2.4/2.5276 GHz, respectively. The scan was acquired with the semi-flexible GEM anterior array. An axial two-dimensional balanced SSFP sequence was used with multiple frames and a frame rate of 1.1 seconds (BW = 250 kHz, pulse repetition time = 4.4 ms, flip angle = 35, resolution = 2.2mm, field-of-view = 50 cm). The data was low-pass filtered and converted to percent modulation units (excluding coils with low means, for which the percent modulation is artificially large). The phase was extracted using the method described in Appendix S1. The low-pass filter cutoff was 2 Hz for respiratory motion (Figure 6A) and 14 Hz for bulk motion (Figure 6C) to better visualize sharp movements.

We further investigated separation between motion types by applying Principal Component Analysis (PCA) to PT and BPT-Rx. We compared the time evolution of three PCs of PT and BPT-Rx for a portion of the data with breathing and bulk motion (Figure 6E). Three PCs were chosen for visualization purposes.

We repeated this experiment with the same volunteer at BPT-Tx frequencies 300/427.6, 800/927.6, 1200/1327.6, and 1800/1927.6 MHz, with simultaneous PT at 127.8 MHz throughout. BPT-Tx transmit powers ranged from 10 to 20 dBm at the antenna, while PT was fixed at −36 dBm at the antenna in order to result in the same received level (explained in Section 2.6). Results from selected coils are shown in Figure S3.

#### 2.6.2 | GEM cardiac motion experiments

To evaluate frequency-dependent cardiac sensing with BPT, we acquired a series of breath-held cardiac scans of a volunteer at BPT-Tx frequencies ranging from 400 MHz to 2.4 GHz (Figure 7), chosen to be the same as the EM simulation (Figure 4). The scans were acquired on the same GEM anterior array coil used in the respiratory experiment but with 16 additional posterior coils (GE Healthcare). BPT-Tx and PT were transmitted simultaneously, with PT at 127.6 MHz and −38 dBm and BPT-Tx ranging from 10 to 17 dBm. The same balanced SSFP sequence was used as in the respiratory experiment; however, scans were acquired with RF excitation and gradients off in order to avoid artifacts (see Section 3.4). BPT-Tx was transmitted using a 2.4 GHz printed circuit-board dipole antenna placed at the top of the bore, while PT was transmitted with a waveform generator (SDG6022X; Siglent Technologies) and a small broadband loop placed on the chest. We used a wideband amplifier and band-stop filter for the BPT-Tx setup (Section 2.4). BPT-Rx and PT were acquired with simultaneous photoplethysmogram (PPG) on the subject’s finger as a reference for signal timing and shape.

The PT/BPT-Rx magnitude signals were low-pass filtered with a cutoff of 25 Hz, mean-subtracted, and normalized to unit variance (Figure 7A). Signals from four coils with the greatest energy in the cardiac frequency range [0.9, 3] Hz were plotted. We further compared the signal characteristics of BPT-Rx and PT (Figure 7B). PT from an empirically chosen coil element was low-pass filtered with a cutoff of 2 Hz, while BPT-Rx was filtered with a cutoff of 25 Hz for best visualization.

We performed an additional cardiac experiment to assess whether rigid contact with the coil was necessary to sense BPT-Rx signal at 2.4 GHz. We measured breath-held cardiac BPT-Rx data while the GEM coil was not in physical contact with the subject. A 2.4 GHz dipole printed circuit-board antenna at the top of the bore transmitted the BPT at 20 dBm and 2.4/2.5278 GHz. The results of this experiment are in Figure S4.

#### 2.6.3 | dBCG Validation

We compared BPT-Rx from breath-held scans of a volunteer to dBCG acquired with a tri-axial accelerometer placed on the chest as well as to PT, ECG, and PPG^45^ (Figure 8). The accelerometer setup (Figure 8B) was the same as in the coil vibration experiment, but with the accelerometer on the subject’s chest. We used a two-dimensional SPGR sequence with RF and gradient excitation turned off, BW = 62.5 kHz and pulse repetition time = 8.7 ms to allow data transfer from the Arduino. As in the cardiac motion experiment, a semi-flexible GEM receiver coil was used, and PPG was measured from the subject’s finger. ECG was also acquired with electrodes on the chest. The accelerometer signals were high-pass filtered with a cutoff of 4Hz, then integrated twice to obtained displacement.

We performed a least-squares fit to compare dBCG and BPT-Rx quantitatively by regressing the multicoil BPT-Rx signals to dBCG measured on the left-right axis. Magnitude BPT data were low-pass filtered with a cutoff of 15 Hz, and all signals were de-meaned before the regression. We denote the resulting signal as BPT-dBCG (Figure 8E).

#### 2.6.4 | AIR coil cardiac motion experiment

To investigate the effect of coil characteristics on BPT-Rx, we additionally measured cardiac signal from a breath-held volunteer scan using a flexible coil array marketed as the AIR coil (GE Healthcare). BPT-Tx and PT were transmitted simultaneously at frequencies 1.8/1.9298 GHz and 129.6 MHz, with transmit powers of approximately 10 and −44 dBm at the antenna, respectively. 129.6 MHz was chosen because PT modulation is empirically more pronounced than at 127.8 MHz yet still in the receiver BW. BPT-Tx was transmitted using the setup described in Figure 3 and a 4G LTE antenna, while PT was transmitted with a waveform generator (SDG6022X; Siglent Technologies) and a small broadband loop, with both BPT and PT transmitters placed on the coil. Data were acquired with RF and gradient excitation turned off. Results from a single coil are shown in Figure S5.

#### 2.6.5 | Head motion experiment

We measured head motion on a volunteer with simultaneous MIMO BPT and PT while acquiring a series of low-resolution three-dimensional images on a rigid 22-channel head-neck coil (GEM HNU; GE Healthcare) that was not touching the subject. The arrangement of coil elements is shown in Figure S7. The goals of the experiment were twofold: first, to evaluate the sensitivity and accuracy of BPT-Rx and PT in detecting head motion, and second, to introduce and evaluate MIMO capability for both BPT-Rx and PT.

For the first goal, we explored whether PT and BPT-Rx could capture the lower-dimensionality of nodding and shaking out of the larger space of possible head positions. We performed a calibration scan and an inference scan. In the calibration scan, the subject moved in a raster fashion to sample all translations and rotations within ±3 mm and ±4 degrees (Figure S6A). During the inference scan, the subject shook their head “no” and nodded their head “yes” (Figure S6B). Both scans were acquired using a 3D SPGR sequence (field of view = 42 × 42 × 27 cm^3^, flip angle = 5, BW = 250 kHz, acceleration = 2.5×). The calibration scan was acquired at ∼1 frame per second with 115 frames, and the inference scan at ∼0.4 frames per second with 50 frames.

The transmit setup consisted of two 2.4 GHz dipole printed circuit-board transmit antennas placed on the top and side of the head coil with cardboard in between the antenna and the coil to prevent detuning (Figure 9A). The frequencies were 2500/2372.39, 2400/2527.78 MHz for BPT-Tx and 127.58, 127.81 MHz for PT with transmit powers of +15 and −42 dBm, respectively. Two SDRs and two dual-channel RF transmit devices (SynthHD; Windfreak Technologies, LLC) were used to transmit all tones. The SDRs and one SynthHD produced two sets of BPT-Tx, while the second SynthHD produced the PTs. Each BPT-Tx chain consisted of a power combiner, narrow-band WiFi amplifier, and high-pass filter (Figure 3). An additional combiner (ZFSC-2–372-S+; Minicircuits) was used to combine the two BPT-Tx tones with a PT, yielding three tones on each antenna.

To evaluate the accuracy of BPT-Rx and PT, we obtained ground-truth motion estimates by registering the low-resolution image timeseries. The registration estimates were Savitzky–Golay filtered (window = 11 samples, order = 4) and compressed to 3 PCs for visualization purposes. The PCs (reg-PCs) explained 99% of the variance of the data. An affine registration, recording translation and rotation, was performed using the SimpleElastix Python package with default settings.^46^

The calibration BPT-Rx and PT data were first preprocessed by averaging over each frame and filtering using a Savitzky-Golay filter (window = 11, order = 4) to reduce noise. Each data set had a dimension of [115 × 22 × 2] corresponding to number of frames, coils and tx antennas, respectively. It was then reshaped to [115 × 44] and compressed to three PCs (Figure S6C,D). The inference BPT-Rx and PT data were preprocessed in the same way and then projected onto the learned PCs. The resulting signals explained ∼95% of the data variance for both BPT-Rx and PT. As in Figure 6E, we plotted each time point in the PC-space (Figure 9D).

To evaluate the MIMO setup quantitatively, we regressed the multicoil BPT-Rx and PT signals to each reg-PC using data from both antennas, top antenna alone, and side antenna alone. A single reg-PC was chosen for plotting (Figure S8), while the correlations with all reg-PCs are reported in Table S3 (bolded values indicate the maximum values). Qualitative plots of the time and PC domain plots using data from both antennas, top alone, and side alone are shown in Figure S9.

### 2.7 | Signal-to-noise ratio

We performed an image SNR comparison to ensure that BPT-Rx does not adversely impact image SNR. A uniform phantom was scanned with BPT-Tx, PT, and no BPT-Tx/PT using a two-dimensional SPGR sequence (pulse repetition time = 34ms, flip angle = 30, resolution = 0.9 mm, BW = 250 kHz, field of view = 50cm) and a panel antenna placed at the top of the bore. The frequencies and powers were 2.4/2.5278 GHz and 17 dBm for BPT-Tx and 127.8 MHz and −40 dBm for PT. BPT-Rx and PT were cropped out of the image. An SNR map was computed using the method developed by Kellman et al.^47^

## 3 | RESULTS

### 3.1 | Coil vibration experiment

**FIGURE 5:**
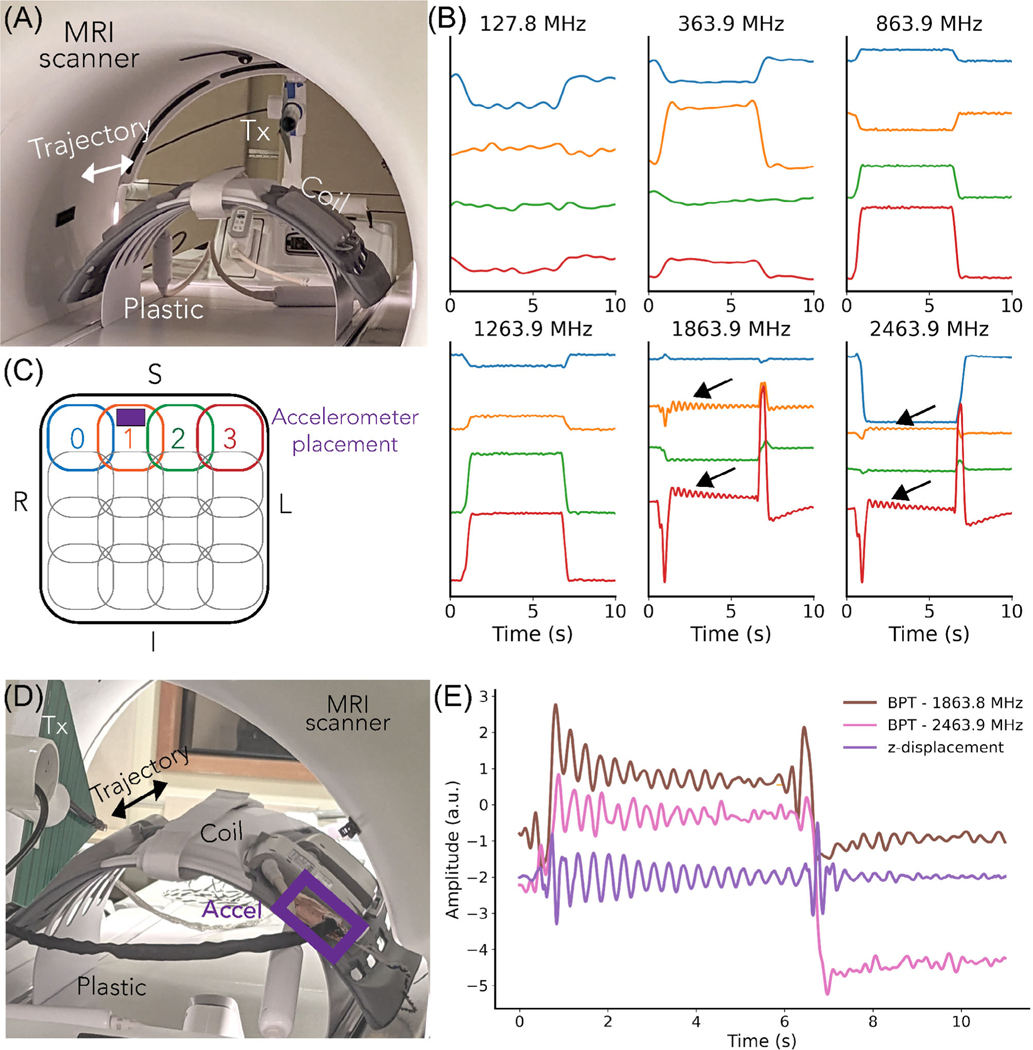
Coil vibration measurement and validation. (A) The cradle was moved at maximum speed and stopped for 5 s. (B) The first period of the PT (top left) and BPT-Rx (remaining plots) is displayed after low-pass filtering. At frequencies >1.2 GHz, BPT-Rx senses the vibration of the coils due to the abrupt motion of the cradle (black arrows). (C) The arrangement of the coils. (D) The setup of the accelerometer experiment, with the accelerometer (accel) indicated by the purple box. (E) The measured BPT-Rx signal at the two highest frequencies vs. z-displacement measured sequentially with accel placed on coil 1. The signals appear to match.

Figure 5B shows the results of the coil vibration experiment, with setup in Figure 5A and physical coil arrangement in Figure 5C. At the two highest frequencies (1.839 and 2.4639 GHz), the coil signals show small fluctuations (black arrows in Figure 5B). We hypothesized that these are vibrations of the coil when the cradle suddenly moves.

We validated this hypothesis by comparing BPT-Rx at the two highest frequencies to displacement measured by the accelerometer. Figure 5E suggests that the displacement (purple) is very similar to BPT-Rx at 1.8 (brown) and 2.4 GHz (pink) in shape and vibration frequency. Moreover, after the cradle is moved in the opposite direction (6–10 s), the vibration appears to be dampened compared to the earlier movement (1–6 s) in both the accelerometer measurement and BPT-Rx. Therefore, it appears that BPT-Rx at high frequencies is able to capture small vibrations of this semi-flexible receiver coil.

### 3.2 | Volunteer experiments

#### 3.2.1 | Respiratory motion experiment

**FIGURE 6:**
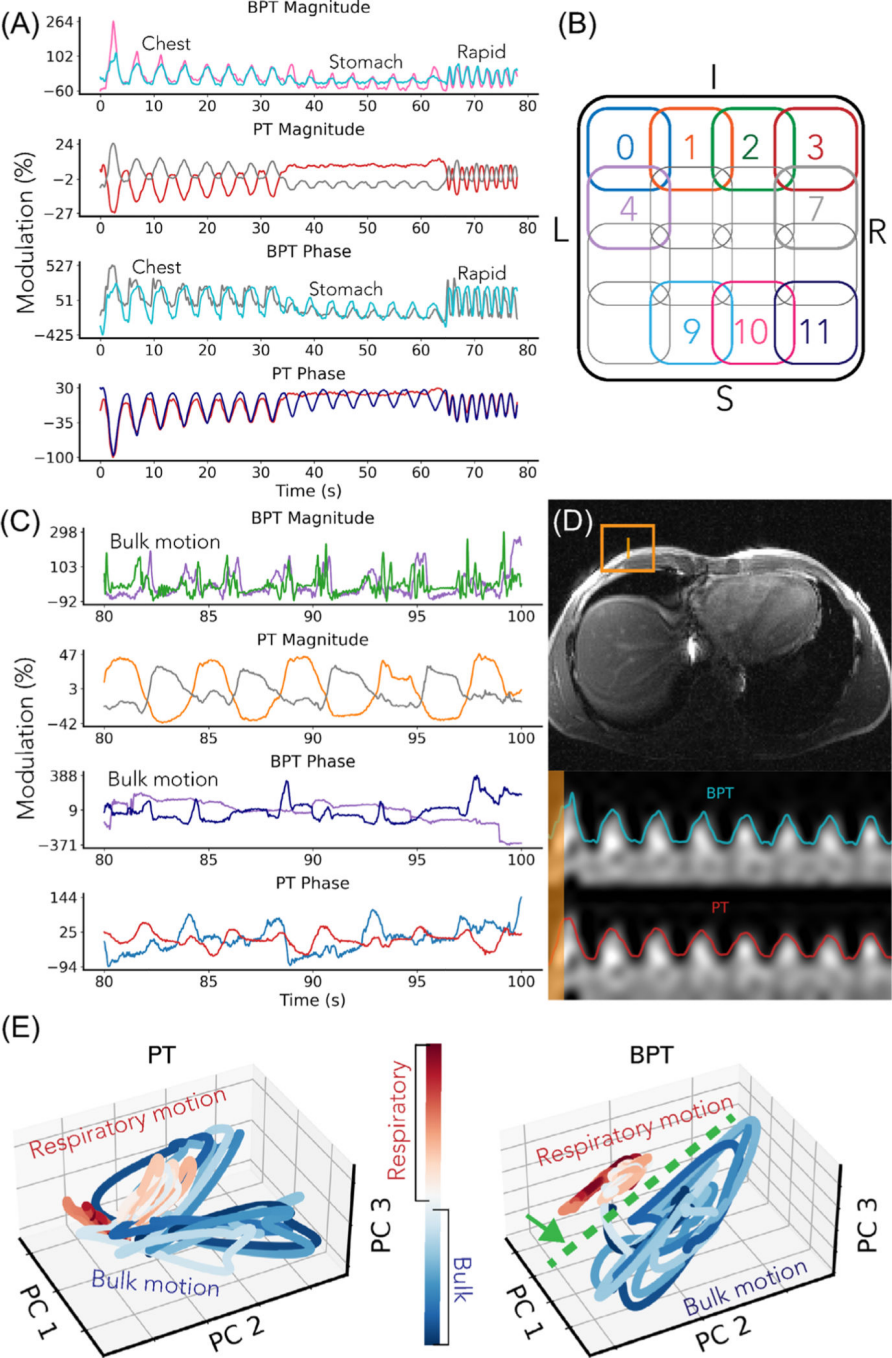
Respiratory motion sensing with BPT and PT. (A) BPT-Rx and PT signals during chest, stomach, and rapid breathing, chosen from the two most modulated coils and displayed in percent modulation units. BPT-Rx modulation is six to seven times greater than PT. (B) Coil arrangement and colors corresponding to (A), (C), and (D). (C) BPT-Rx magnitude and phase modulation (first and third row) and PT magnitude and phase modulation (second and last row) during bulk motion. BPT-Rx modulation is larger and sharper than the PT. (D) BPT-Rx (top) and PT (bottom) magnitude overlaid on a patch of the image (orange box) for coils 9 and 3, respectively. Both appear to match the displacement of the patch qualitatively. (E) The time evolution of the three main PCs of the PT and BPT-Rx during breathing and bulk motion. The green arrow and line indicate a separating plane between breathing and bulk motion.

The respiratory motion experiment suggests that BPT-Rx is sensitive to changes in breathing and bulk motion (Figure 6). Figure 6A displays the two most modulated coils from BPT-Rx and PT, each scaled within their maximum and minimum percent modulation. The numbered coils are physically arranged as in Figure 6B. We compared the range (max–min) of BPT-Rx and PT modulations. BPT-Rx is 6.3× more modulated than PT in magnitude and 7.3× in phase during breathing. When overlaid on a patch of the image over time, both BPT-Rx (top) and PT (bottom) breathing signals appear comparable (Figure 6D).

BPT-Rx is also 4.3× more modulated than the PT in the bulk motion portion of the experiment in magnitude and 3.2× in phase (Figure 6C). While the BPT-Rx magnitude shows much sharper peaks for bulk motion than breathing, the PT magnitude is smoother, similar to the breathing signal. Thus, Figure 6A,C suggests that BPT-Rx can achieve greater motion sensitivity than PT, and that bulk motion appears noticeably different from respiration.

Figure 6E further demonstrates that breathing is better separated from bulk motion in BPT-Rx PCs (right) compared to PT PCs (left), with the green dashed line indicating a separating plane. Separation in the PC feature space may be a good metric to distinguish between motion types. For instance, after a training period, this information could be used to reject outliers or prospectively acquire data only during respiratory motion.

We found that BPT-Rx signals from some coils over a broad BPT-Tx frequency range have multiple peaks. The simulation (Figure 4) suggested that standing wave patterns with multiple peaks and nulls are created when transmitting into an empty bore. In a setting with a human subject in the bore, the body modulates the standing wave based on its conductive properties, and this standing wave changes with motion; thus, the setting is much more complex. Figure S3A shows manually chosen coil signals from the respiratory experiment with multiple peaks across a broad range of BPT-Tx frequencies (300/427.6, 800/927.6, 1800/1927.6, 2400/2527.6 MHz), with corresponding coil positions in Figure S3B. This result further supports the standing wave mechanism.

#### 3.2.2 | GEM cardiac motion experiments

**FIGURE 7:**
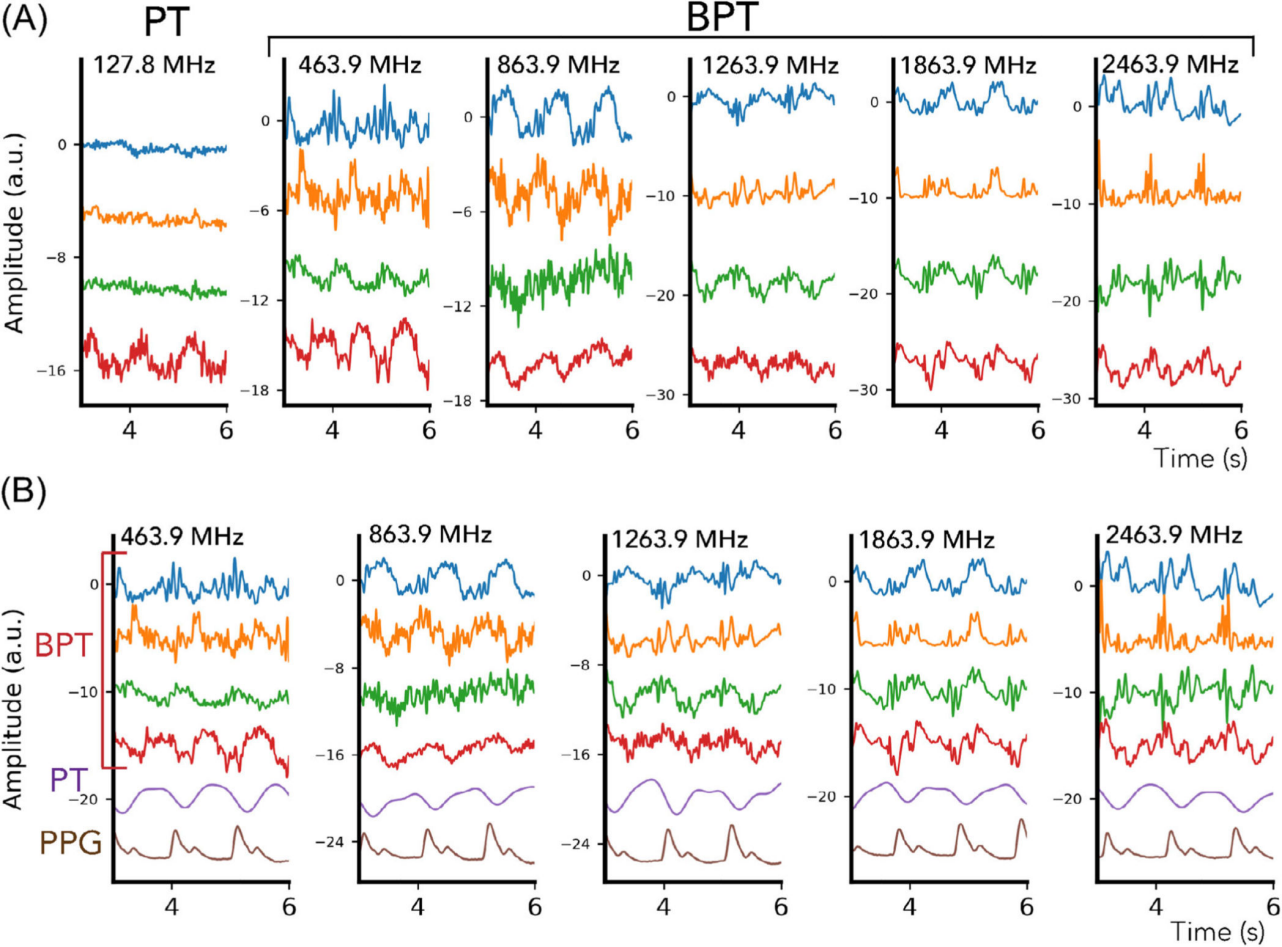
Cardiac motion sensing with PT and BPT. Cardiac PT and BPT-Rx signals were obtained simultaneously from a healthy volunteer during breath-held scans across BPT-Tx frequencies. (A) PT and BPT-Rx signals from the four coils with the greatest energy in the cardiac frequency range after low-pass filtering with a cutoff of 25 Hz, mean-subtraction, and normalization to unit variance. (B) Low-pass filtered PT and BPT-Rx signals, along with simultaneous photoplethysmogram (bottom). The filter cutoffs were 2 Hz for PT and 25 Hz for BPT. The cardiac PT/BPT-Rx signal appears smooth at frequencies <1.2 GHz but contains sharp peaks for higher frequencies, suggesting that BPT-Rx may be more sensitive to blood volume changes at lower frequencies and surface vibrations at higher frequencies.

On the GEM semi-flexible coil array, BPT-Rx at low frequencies appears to reflect blood volume changes, while BPT-Rx at high frequencies appears more sensitive to surface vibrations of the body. Figure 7A shows PT and BPT-Rx magnitudes from the four coils with the greatest energy in the cardiac frequency range. PT cardiac signal appears noisier and contains a single peak for each heartbeat, a signal shape that arises from blood volume changes.^34^ However, the BPT-Rx signals at high frequencies (1.8 and 2.4 GHz) are cleaner and appear to contain multiple peaks. Figure 7B further shows the differences between PT, BPT-Rx, and PPG acquired simultaneously. At lower frequencies (<1.2 GHz), BPT-Rx changes smoothly. At higher frequencies (≥1.2 GHz), BPT-Rx contains multiple sharp peaks. It appears that the sensing mechanism is different—as explained in the next section, we hypothesize that BPT-Rx at high frequencies captures dBCG, which measures millimeter-scale vibrations of the body due to the ballistic forces of blood. Given that intermediate frequencies (464 and 864 MHz) may contain more than one peak per cycle but have a similar shape to PT, it is possible that the cardiac signal arises both from both blood volume changes and dBCG. The improved quality of the signal at high frequencies suggests that BPT-Rx cardiac signal can be extracted from the raw data with minimal processing, which makes BPT a promising candidate for real-time cardiac gating applications.

However, Figure S4 suggests that rigid contact with the subject may be required to obtain these BPT-Rx results at high frequencies. When the coil is elevated above the subject (Figure S4B), the sharp peaks do not appear as visible (Figure S4D). There is still some cardiac modulation, but it appears weaker. We hypothesize the reasons for this in Section 3.2.4.

#### 3.2.3 | dBCG validation

**FIGURE 8:**
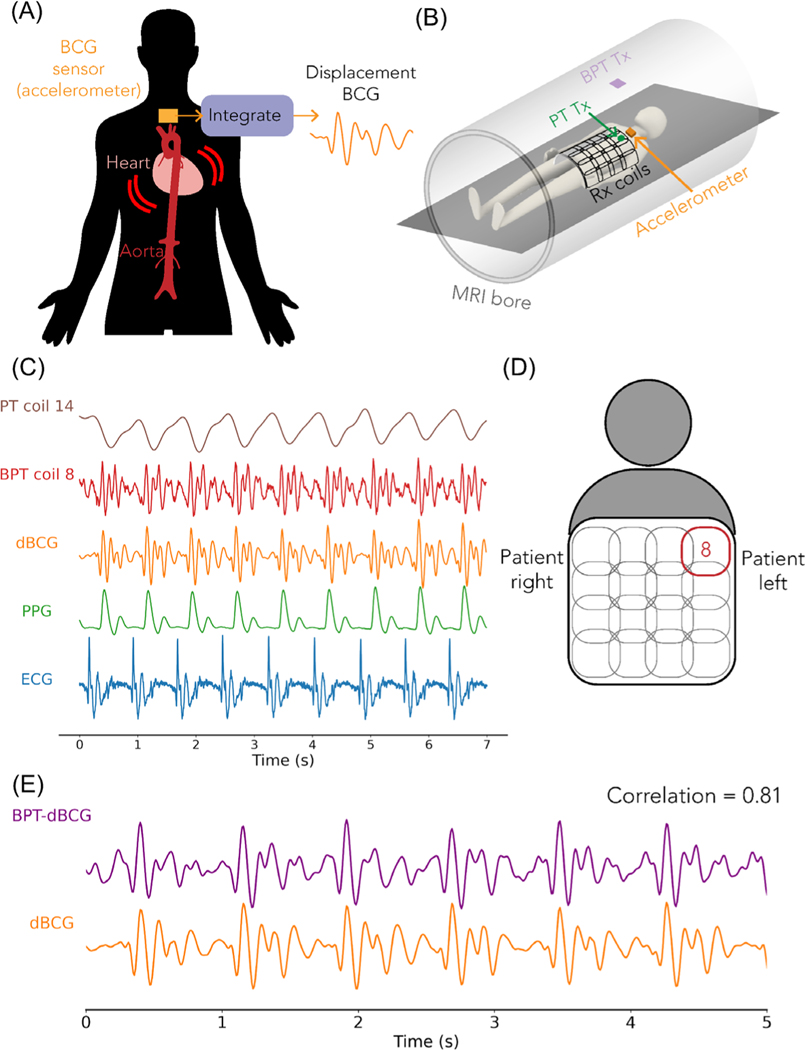
dBCG measurement and validation with BPT. (A) Displacement BCG (dBCG) measures the recoil of the body due to the ballistic forces of blood through the aorta. It was measured with a tri-axial accelerometer, then integrated twice to obtain displacement. (B) Placement of the BPT tx antenna, PT tx antenna, and the accelerometer in the bore.^45^ (C) Low-pass filtered PT (cutoff = 3 Hz), BPT-Rx signal from a single coil versus computed dBCG, PPG, and ECG. The first wave of BPT-Rx and dBCG appear later than ECG but earlier than PPG. (D) Physical location of the BPT coil corresponding to (C). (E) BPT signals were regressed to dBCG, then low-pass filtered; this is denoted as BPT-dBCG. BPT-dBCG correlates strongly with dBCG (correlation = 0.81).

Cardiac signals acquired by BPT-Rx at 2.4/2.5278 GHz highly correlate with dBCG^45,48,49^ (Figure 8). This suggests not only that BPT-Rx at microwave transmit frequencies is sensitive to very small vibrations but also that BPT-Rx could offer information about the cardiac cycle that is complementary to MRI. dBCG can be measured in many ways, including fiber-optic sensors,^50^ pneumatic sensors,^48^ cameras,^51^ doppler radar,^52^ or accelerometers.^51^

Figure 8C demonstrates the timing of the raw BPT-Rx signal from the coil in Figure 8D relative to low-pass filtered PT (cutoff = 3 Hz), dBCG, PPG, and ECG signals. The timing and peaks of the BPT-Rx signal qualitatively match dBCG. Quantitatively, the regressed BPT-dBCG has a strong correlation of 0.81 with dBCG (Figure 8E).^45^ We hypothesize that BPT senses mechanical vibrations; therefore, BPT-dBCG may be enhanced due to the stiffness of the receiver array and contact with the subject.

#### 3.2.4 | AIR coil cardiac experiment

One caveat, however, is that dBCG may not be visible with BPT-Rx on all coils. The AIR coil experimental results are shown in Figure S5. The top subplot shows percent modulation of BPT-Rx and PT relative to the mean. While BPT-Rx is approximately twice as modulated as PT, the modulation is still within ±1%, which is much smaller than the level of modulation in BPT-dBCG (≈ 30%^45^). Moreover, the signal shape and timing appear very similar between PT and BPT-Rx. We hypothesize that BPT-dBCG is visible in the experiments with the GEM coil because of its mechanical stiffness, which amplifies vibrations of the body when touching it. In contrast, the AIR coil is flexible and may dampen these vibrations. Figure S4 further supports this hypothesis—when the GEM coil is not in physical contact with the subject and thus not vibrating, BCG does not appear visible.

The cardiac results suggest that BPT may be influenced by blood volume changes as well as surface vibrations of the body and the coil. The relative contribution of each of these mechanisms appears to depend on the stiffness of coil, the level of contact with the subject, and the transmit frequencies.

#### 3.2.5 | Head motion experiment

**FIGURE 9:**
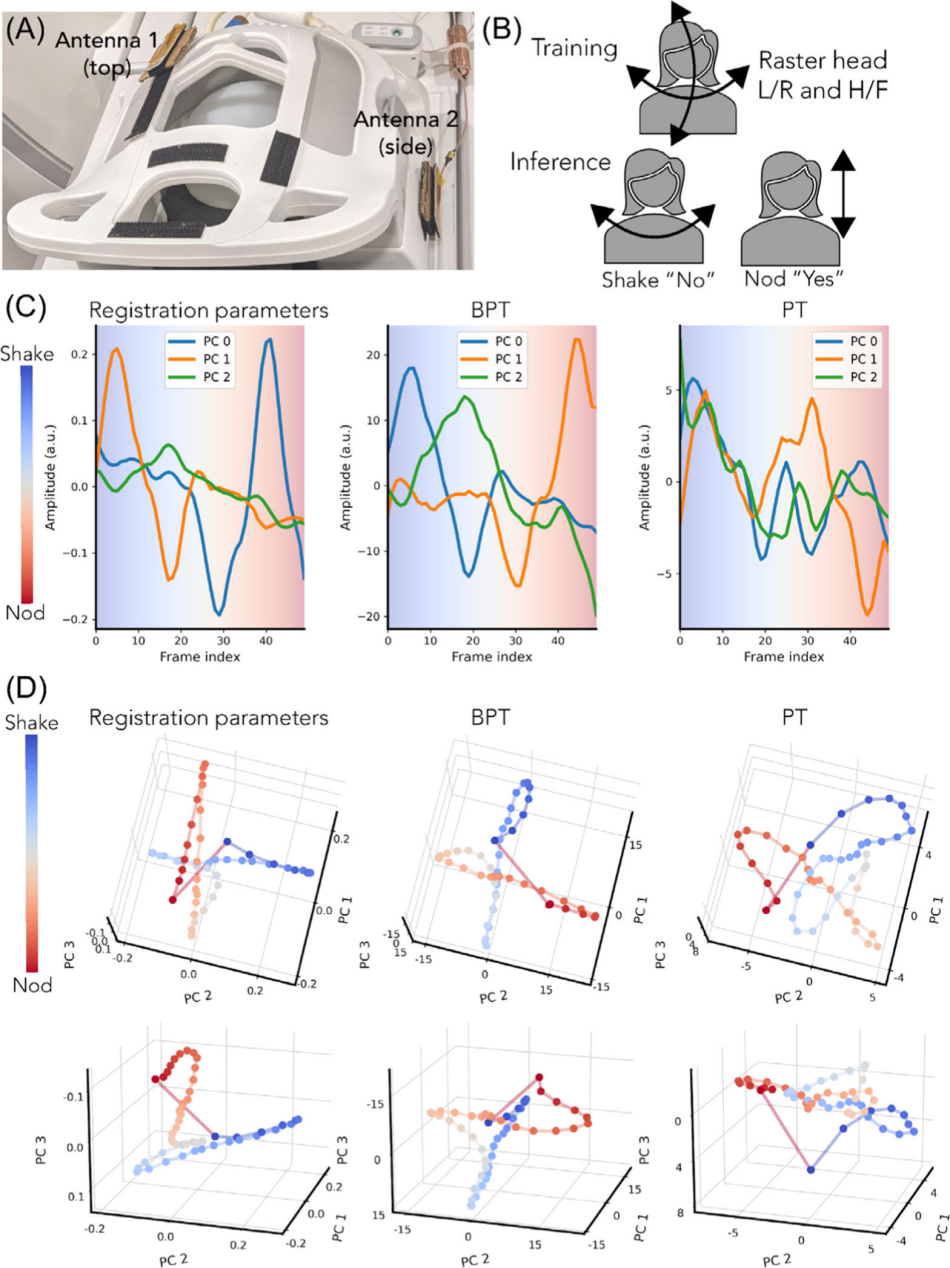
MIMO head motion sensing with BPT. (A) Two antennas were placed at the top and side of the head coil, each transmitting two BPT-Tx tones and one PT. (B) During a calibration or training scan, the volunteer rotated and translated their head in a raster fashion. During an inference scan, they shook “no” (left-right) and nodded “yes” (up-down). (C) The three main principal components (PCs) of the ground-truth registration parameters (reg-PCs), BPT-Rx and PT plotted on the same scale versus time and (D) in the PC feature space viewed from two different angles (top, bottom). The two head motions (shake “no,” nod “yes”) show the same “x” shape in reg-PCs and BPT-Rx, with the crossing point corresponding to the common head position between nodding and shaking. However, PT does not show the same structure or smoothness.

Figure 9C shows the three PCs for the registration parameters (reg-PCs), BPT-PCs, and PT-PCs over time, while Figure 9D shows the same data in the PC space from two viewing angles. The smoothness of the BPT-PC curve in Figure 9C suggests that the BPT-PCs may be less noisy than PT. In Figure 9D, the BPT-PCs overall look very similar to the reg-PCs. Both BPT-PCs and reg-PCs show a crossing behavior that corresponds to the position of the head between nodding and shaking. Moreover, both show smooth trajectories for nodding and shaking. However, the PT-PCs are noisier, with less clear structures to distinguish nodding from shaking.

Quantitatively, MIMO appears to have benefits for BPT-Rx and PT: the regressed BPT-Rx and PT (Figure S8, Table S3) computed with data from both antennas have greater correlation with the reg-PCs than regressed BPT-Rx/PT using data from a single antenna. Regressed BPT-Rx (Figure S8B) has consistently higher correlation than PT (Figure S8A) over all reg-PCs (Table S3). Figure S9 shows the time and PC-domain plots for the top and side antenna data alone, showing qualitative differences between the two datasets. These results suggest that MIMO-BPT has the potential for accurate quantitative rigid motion correction.

### 3.3 | Signal-to-noise ratio

**FIGURE 10:**
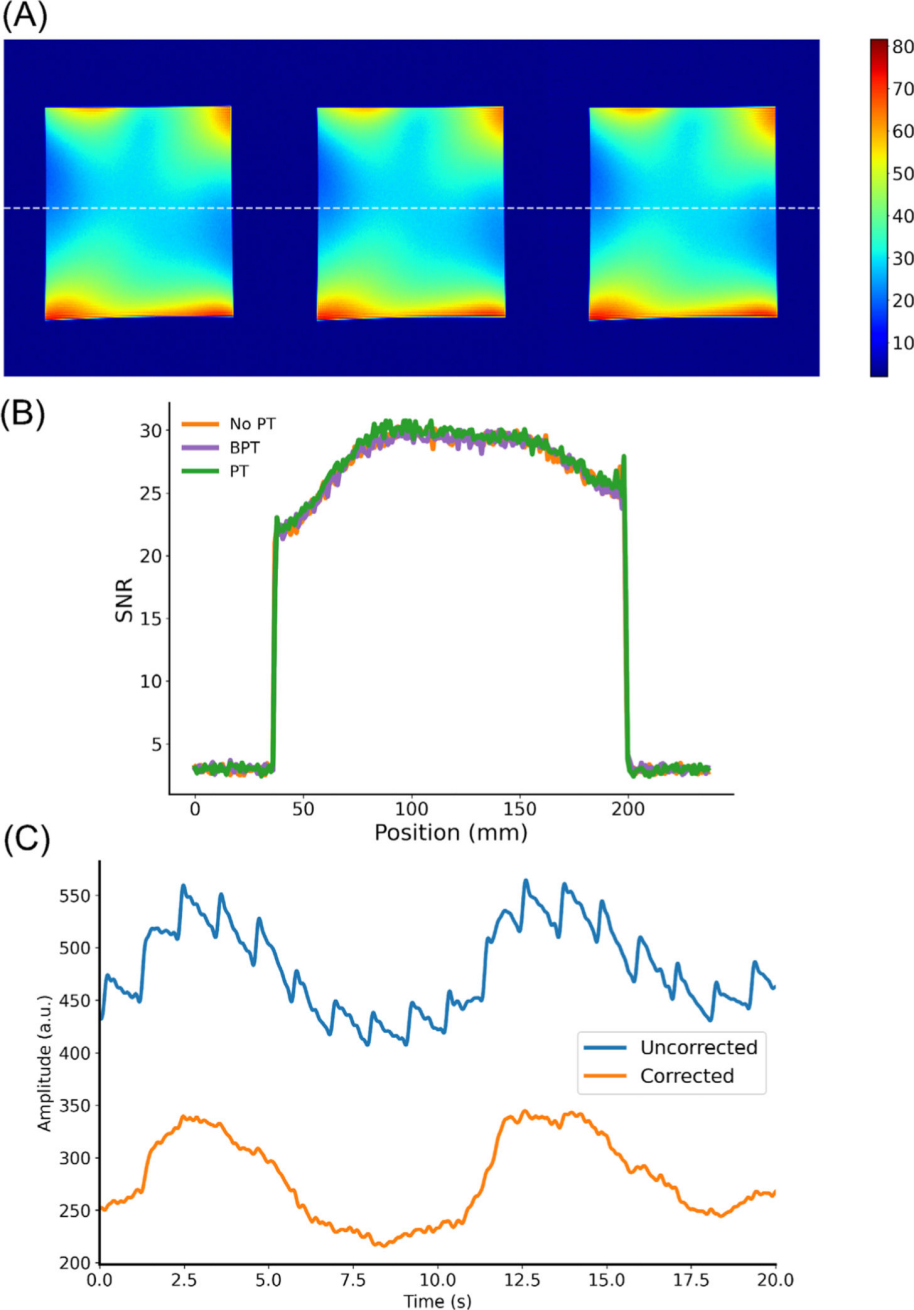
Signal-to-noise ratio (SNR) and artifacts. (A) Image SNR maps were computed in three acquisitions with no PT, BPT, and PT using a uniform phantom with a 32-channel anterior/posterior GEM array coil. (B) Line plots through the center of the maps, indicated by the dashed line. (C) Switching gradient fields can cause the antenna to vibrate, resulting in artifacts in BPT-Rx (top, blue), which was measured with BPT-Tx frequencies of 2.4/2.5478 GHz. These can be corrected (bottom, orange) by a linear fit, as described in the Appendix S1.

The SNR maps (Figure 10A) and line profiles (Figure 10B) are nearly identical with and without BPT or PT. This suggests that PT and BPT-Rx have no adverse impact on SNR. For the transmit power levels used in the volunteer experiments and SNR maps, there is no visible receiver gain compression.

### 3.4 | Vibration artifacts

The high sensitivity of the BPT also causes it to be sensitive to other vibrations of the system (Figure 10C). We hypothesize that the antenna and receiver coils are caused to vibrate by mechanical and electrical coupling to the MRI scanner. Mechanically, structures in the scanner vibrate due to Lorentz forces on the coil windings from the static magnetic field $\left({\text{B}}_{0}\right)$. If the antenna or receiver array is placed on any vibrating surface, it will also vibrate. Electrically, the antenna and coils may experience Lorentz forces due to induced eddy currents from the switching gradient fields, causing them to vibrate. The electrical coupling occurs only if the antenna and coils are placed inside the MRI bore. This artifact can be mitigated by filtering, placing the antenna outside the bore, or dampening its vibration. It may also be approximated by using a linear fit per-coil and subtracting the estimate from the data, which is described in the Appendix S1.

## 4 | DISCUSSION

In this paper, we present BPT, a simple system exploitation that enables simultaneous RF motion sensing with the MRI data acquisition. BPT has the potential to operate at any frequency and in any MRI scanner independent of field strength. Moreover, the transmitter setup is simple to implement. BPT uses RF standing waves to obtain percent modulation that can be more than 5 times greater than PT. By removing the requirement that the transmitted RF be tied to the Larmor frequency, BPT generalizes RFC methods and opens up new possibilities for frequency-dependent motion detection. Our preliminary results show that BPT can separate respiratory from bulk motion, capture dBCG at microwave frequencies on a semi-rigid coil, and operate as a MIMO system.

Simulations suggest that BPT samples standing wave patterns in the bore (Figure 4). These simulations agree well with experimental results. Differences between simulation and experiment could be due to antenna characteristics and additional structures in the bore, such as the cradle, cables, and additional coils in the bed, which are not modeled in the simulation for simplicity. There may also have been unsuppressed common-mode parasitics, which could have radiated from the transmit cable and caused additional reflections. Accurate modeling of the human body may be necessary to simulate the behavior of BPT ona human subject.

The simulation considers only the magnitude of the flux, |Φ|. Though the voltage is the time derivative of the flux, because the fields are complex exponentials in time, the voltage is directly proportional to the flux, that is, $V(t)=e^{j\omega t}\text{Φ}(t)$. The scaling does not affect the magnitude of the signal. Therefore, we omitted it. We chose to compare only |Φ| between simulation and experiment because there are possible contributions to the phase that were not part of the simulation, such as AM-PM modulation.^39^ Due to these effects, the level of modulation in the measured phase is greater than expected in all experiments.

Volunteer experiments suggests that BPT’s sensitivity to motion depends on the transmit frequencies, antenna placements, and coil characteristics. Cardiac BPT-Rx on the semi-flexible GEM coil correlates strongly with dBCG acquired simultaneously using an accelerometer when the coil is moving with the subject (Figure 8). However, dBCG does not appear visible in BPT-Rx on the flexible AIR coil (Figure S5), nor on the GEM coil when it is not in contact with the subject (Figure S5). BPT-Rx on the AIR coil appears similar to PT in signal shape, timing, and level of modulation. The head motion experiment used a coil that was not in rigid contact with the subject, yet still showed high correlation with ground-truth motion estimates from image registration (Table S3). The contributions to BPT-Rx are thus complex and require further exploration to characterize fully.

We have demonstrated that BPT can be scaled to multiple antennas (Figure 9), and MIMO may allow the ability to separate and learn different motion trajectories. MIMO-BPT appears more similar to the reg-PC curves, and achieves high correlation (>0.97) with the reg-PCs when regressed; however, further analysis is required to investigate whether it provides benefits for quantitative head motion correction compared to a single BPT-Tx or PT.

In all experiments, we compared BPT-Rx to PT and common peripheral sensors (ECG and PPG). We could have additionally compared BPT-Rx to other RFC methods such as reflected power from a parallel transmit array^23^ or the noise navigator.^22^ However, our MRI system does not have a parallel transmit array available. Moreover, the noise navigator is passive and thus has inherently lower SNR compared to active methods such as PT.^33^ Therefore, we determined that the most reasonable comparison would be to PT.

BPT exploits a nonlinear property of the preamps; however, this nonlinearity may differ between coils and systems, and thus be a potential barrier to general implementation. Some array coil electronics may have inductors in the receive path before the preamp. Then, frequencies at the GHz range will be attenuated, and sensing BPT-Rx may not be possible without altering the coils. We also chose to focus on second order intermodulation (i.e., $f_{\text{BPT}}=f_{2}-f_{1}$) in this work. A higher order may allow for a larger BW of intermodulation; however, this may require greater transmit power to overcome attenuation by the receiver chain.

## 5 | CONCLUSION

BPT offers a rich dataset of motion information. Obtaining dBCG measurements with BPT could characterize cardiac function simultaneously to the MRI exam.^53,54^ BPT has the ability to distinguish between motion types, offering the potential for improved motion correction. Preliminary work has demonstrated the utility of BPT for retrospective head motion correction.^29^ Using BPT with the MRI system has many other potential applications, including cardiac and respiratory gating^31^; motion sensing for low-field systems, in which body impedance changes are minimal^55,56^; sensing motion deep in the body such as fetal motion^57^; or using multiple transmitters to reconstruct microwave images.^58^ For example, researchers in Shanghai Jiao Tong University have implemented BPT on a low-field 0.25T MRI system to sense and correct for respiratory motion.^55^ The simplicity of implementation could enable widespread adoption in many MRI systems.

## Supplementary Material

Supinfo **Figure S1.** Intermodulation measurements on a preamp. The strength of the intermodulation product (IMD) was measured for two tones at 2.4 GHz (left) and 2.5278 GHz (right) on a preamplifier interface box for custom receiver coils (Clinical MR Solutions, LLC). Using a spectrum analyzer (FieldFox N9918A; Keysight Technologies), the input power was swept from −14 to +2dBm, and the power of the IMD was measured, along with the output power at 2.4 and 2.5278 GHz (“fundamental”). The second-order intercept point (IP2) where the lines cross was extrapolated based on fitting lines to the fundamental and IMD data. The measurements suggest that it is possible to obtain an IMD power close to that of the MR signal (>−70 dBm) with little BPT-Tx power (−10 dBm). Thus, intermodulation in the preamp is the likely mechanism of BPT-Rx, and there may not be significant gain suppression so far from the IP2 point.
**Figure S2.** Bulk motion in the respiratory experiment. Bulk motion estimates from the respiratory experiment, with rotation angle on the left, and displacements on the right. The estimates were obtained by registering the images using SimpleElastix,^46^ a rigid transformation, and default rigid registration options (mutual information as the metric; multiresolution registration; stochastic gradient descent). The rotation was within ±3 degrees about the head-foot axis, and the displacement was between −15 and 5 mm in the left-right and anterior-posterior axes.
**Figure S3.** BPT-Rx respiratory signal over frequency. A volunteer performed different breathing types (chest breathing, stomach breathing, rapid-shallow breathing, and breathing with simultaneous bulk motion of the chest). The experiment was repeated at BPT-Tx frequencies of 300/427.6, 800/927.6, 1200/1327.6, 1800/1927.6, and 2400/2527.6 MHz. (A) BPT-Rx signals with multiple peaks were manually chosen from each experiment, with coil positions in (B). There appears to be at least one coil signal with multiple peaks for each of the frequencies, except for 1200/1327.6 MHz. These peaks could be due to the shape of the BPT-Tx standing wave patterns.
**Figure S4.** Rigidly contacting versus elevated GEM coil cardiac experiment. Breath-held cardiac BPT-Rx signals were acquired on a volunteer with (A) the GEM anterior array (AA) coil placed on the chest and (B) elevated from the chest using a plastic support with BPT-Tx at 2.4/2.5278 GHz. BPT signals from selected AA coils in (C) the normal configuration and (D) the elevated configuration. The elevated signals show lower cardiac modulation, and perhaps no longer a dBCG signal.
**Figure S5.** AIR coil results. BPT-Rx and PT were acquired simultaneously in a breath-held scan with RF excitation and gradients off using an abdominal AIR coil. The BPT-Tx frequencies were 1.8/1.9298 GHz, and PT was 129.6 MHz. Top: BPT-Rx and PT are plotted for a single coil in percent modulation units after being low-pass filtered with a cutoff of 5 Hz. Bottom: The same BPT-Rx and PT data after band-pass filtering between 0.5 and 5Hz. Both appear comparable in terms of signal shape and modulation level.
**Figure S6.** Motion parameters during the MIMO head motion experiment. In the calibration scan, the subject attempted to capture the full rigid parameter space (i.e., all possible translations and rotations within an approximate range of ±3mm and ±4 degrees) by moving in a raster fashion. In the inference scan, they shook their head “no” and nodded “yes.” PT and BPT-Rx data were combined across antennas and averaged over frames. Rigid motion parameters were estimated from registering the images from (A) the calibration scan and (B) the inference scan after Savitzy-Golay filtering (window size = 11, order = 4). PT (top) and BPT (bottom) from the four most modulated coils are plotted from the (C) the calibration scan and (D) the inference scan after Savitzky-Golay filtering (window size = 11, order = 4).
**Figure S7.** GEM head coil arrangement. (A) Rendering of the GEM Head and Neck Unit (HNU) coil used for the head motion experiment. (B) Approximate physical arrangement of the 22 coil elements. Elements 0, 1, and 2 belong to the posterior array integrated into the scanner bed.
**Figure S8.** BPT-Rx and PT regression to reg-PC 1. Filtered and concatenated data were regressed to reg-PC 1 for (A) BPT-Rx and (B) PT data. The top row is for data from the top antenna, the second row for the side antenna, and the bottom row for both. Pearson correlation coefficient is reported for each subplot. The correlation is higher for PT and BPT-Rx when using data from both antennas and is higher for BPT-Rx than for PT.
**Figure S9.** PCs with different antennas versus combined. (A) BPT and PT PCs computed only from the antenna at the top. The PCs from registration were unchanged. (B) BPT and PT PCs from the top antenna in the PC space. (C) BPT and PT PCs computed from the side antenna versus time and (D) in the PC space.
**Table S1.** Type and placement of BPT and PT antennas: this table shows the type and placement of antennas for BPT and PT. Double dashes (“–”) indicate that the antenna or placement was the same, for example, the same log periodic antenna was used to transmit both BPT and PT.
**Table S2.** 2.4GHz BPT hardware.
**Table S3.** PC correlation values. We regressed the multicoil BPT-Rx and PT signals to each reg-PC using data from the top antenna alone, the side antenna alone, and both antennas. We report the Pearson correlation coefficient of each reg-PC with each regressed BPT-Rx/PT. Bolded values indicate the maximum correlation value with each reg-PC.

## Data Availability

The datasets generated and analyzed during the current study are available via https://doi.org/10.5281/zenodo.10967226. The code is publicly available on https://github.com/mikgroup/bpt_paper, with the latest commit hash as 0e80ed0.

## References

[1] Sadigh G, Applegate KE, Saindane AM. Prevalence of Unanticipated events associated with MRI examinations: A benchmark for MRI quality, safety, and patient experience. J Am Coll Radiol. 2017;14:765–772. 28356198 10.1016/j.jacr.2017.01.043

[2] Runge VM, Richter JK, Heverhagen JT. Motion in magnetic resonance: new paradigms for improved clinical diagnosis. Invest Radiol. 2019;54:383–395. 30946182 10.1097/RLI.0000000000000566

[3] Zaitsev M, Maclaren J, Herbst M. Motion artifacts in MRI: a complex problem with many partial solutions. J Magn Reson Imaging. 2015;42:887–901. 25630632 10.1002/jmri.24850 PMC4517972

[4] Maclaren J, Herbst M, Speck O, Zaitsev M. Prospective motion correction in brain imaging: a review. Magn Reson Med. 2013;69:621–636. 22570274 10.1002/mrm.24314

[5] Godenschweger F, Kägebein U, Stucht D, Motion correction in MRI of the brain. Phys Med Biol. 2016;61:R32–R56. 26864183 10.1088/0031-9155/61/5/R32 PMC4930872

[6] Pipe JG. Motion correction with PROPELLER MRI: application to head motion and free-breathing cardiac imaging. Magn Reson Med. 1999;42:963–969. 10542356 10.1002/(sici)1522-2594(199911)42:5<963::aid-mrm17>3.0.co;2-l

[7] Prieto C, Batchelor PG, Hill D, Hajnal JV, Guarini M, Irarrazaval P. Reconstruction of Undersampled dynamic images by Modeling the motion of object elements. Magn Reson Med. 2007;57:939–949. 17457881 10.1002/mrm.21222

[8] Huttinga NR, van den Berg CAT, Luijten PR, Sbrizzi A. MR-MOTUS: model-based non-rigid motion estimation for MR-guided radiotherapy using a reference image and minimal K-space data. Phys Med Biol. 2020;65:65. 10.1088/1361-6560/ab554a 31698354

[9] Madore B, Hess AT, van Niekerk AMJ, External hardware and sensors, for improved MRI. J Magn Reson Imaging. 2023;57:690–705. 36326548 10.1002/jmri.28472 PMC9957809

[10] Huttinga NR, Bruijnen T, van den Berg CA, Sbrizzi A. Gaussian processes for real-time 3D motion and uncertainty estimation during MR-guided radiotherapy. Med Image Anal. 2023;88:102843. 10.1016/j.media.2023.102843 37245435

[11] Eschelbach M, Aghaeifar A, Bause J, Comparison of prospective head motion correction with NMR field probes and an optical tracking system. Magn Reson Med. 2019;81:719–729. 30058220 10.1002/mrm.27343

[12] Stam MK, Crijns SP, Zonnenberg BA, Navigators for motion detection during real-time MRI-guided radiotherapy. Phys Med Biol. 2012;57:6797–6805. 23032581 10.1088/0031-9155/57/21/6797

[13] Cheng JY, Zhang T, Ruangwattanapaisarn N, Free-breathing pediatric MRI with nonrigid motion correction and acceleration. J Magn Reson Imaging. 2015;42:407–420. 25329325 10.1002/jmri.24785 PMC4404177

[14] Odille F, Cîndea N, Mandry D, Pasquier C, Vuissoz PA, Felblinger J. Generalized MRI reconstruction including elastic physiological motion and coil sensitivity encoding. Magn Reson Med. 2008;59:1401–1411. 18421689 10.1002/mrm.21520

[15] McClelland JR, Hawkes DJ, Schaeffter T, King AP. Respiratory motion models: a review. Med Image Anal. 2013;17:19–42. 23123330 10.1016/j.media.2012.09.005

[16] White N, Roddey C, Shankaranarayanan A, PROMO: real-time prospective motion correction in MRI using image-based tracking. Magn Reson Med. 2010;63:91–105. 20027635 10.1002/mrm.22176 PMC2892665

[17] Maclaren J, Armstrong BS, Barrows RT, Measurement and correction of microscopic head motion during magnetic resonance imaging of the brain. PLoS One. 2012;7:3–11. 10.1371/journal.pone.0048088 PMC3492340 23144848

[18] van der Kouwe A. Motion Artifacts and Correction in Neuro MRI. Vol 4. Elsevier Inc.; 2021.

[19] Frost R. k-space navigators. Adv Magn Reson Technol Appl. 2023;6:209–224.

[20] Buikman D, Helzel T, Röschmann P. The rf coil as a sensitive motion detector for magnetic resonance imaging. Magn Reson Imaging. 1988;6:281–289. 3398735 10.1016/0730-725x(88)90403-1

[21] Roemer PB, Edelstein WA, Hayes CE, Souza SP, Mueller OM. The NMR phased array. Magn Reson Med. 1990;16:192–225. 2266841 10.1002/mrm.1910160203

[22] Andreychenko A, Raaijmakers AJ, Sbrizzi A, Thermal noise variance of a receive radiofrequency coil as a respiratory motion sensor. Magn Reson Med. 2017;77:221–228. 26762855 10.1002/mrm.26108

[23] Hess AT, Tunnicliffe EM, Rodgers CT, Robson MD. Diaphragm position can Be accurately estimated from the scattering of a parallel transmit RF coil at 7 T. Magn Reson Med. 2018;79:2164–2169. 28771792 10.1002/mrm.26866 PMC5836958

[24] Speier P, Fenchel M, Rehner R. PT-Nav: a novel respiratory navigation method for continuous acquisitions based on modulation of a pilot tone in the MR-receiver. ESMRMB 2015, 32nd Annual Scientific Meeting, Edinburgh, UK, 1–3 October: Abstracts, Thursday, Magnetic Resonance Materials in Physics, Biology and Medicine. 2015;28:1–135.

[25] Bacher M. Cardiac Triggering Based on Locally Generated Pilot-Tones in a Commercial MRI Scanner: A Feasibility Study. Master Thesis. Graz University of Technology. 2017 https://diglib.tugraz.at

[26] Thiel F, Hein M, Schwarz U, Sachs J, Seifert F. Combining magnetic resonance imaging and Ultrawideband radar: a new concept for multimodal biomedical imaging. Rev Sci Instrum. 2009;80:1–10. 10.1063/1.3065095 19191450

[27] Wang H, Li Y, Xia X, Hu L, Zhao J, Chen Q. Non-contact respiratory triggering for clinical MRI using frequency modulated continuous wave radar. Medical Imaging 2021: Physics of Medical Imaging. SPIE; 2021:268–275.

[28] Li C, Lubecke VM, Boric-Lubecke O, Lin J. A review on recent advances in Doppler radar sensors for noncontact healthcare monitoring. IEEE Trans Microwave Theory Tech. 2013;61:2046–2060.

[29] Huttinga N, Anand S, van den Berg AT, Sbrizzi A, Lustig M. Three-dimensional rigid head motion correction using the beat pilot tone and Gaussian processes. Proc Intl Soc Mag Reson Med. 2023;31:1019.

[30] Anand S, Lustig M. Beat pilot tone: exploiting preamplifier intermodulation of UHF/SHF RF for improved motion sensitivity over pilot tone navigators. Proc Intl Soc Mag Reson Med. 2021;29:568.

[31] LamarBruno K, Anand S, Lustig M. Cardiac and respiratory-resolved image reconstruction with the beat pilot tone. Proc Intl Soc Mag Reson Med. 2022;30:4446.

[32] Vahle T, Bacher M, Rigie D, Respiratory motion detection and correction for MR using the pilot tone: applications for MR and simultaneous PET/MR examinations. Invest Radiol. 2020;55:153–159. 31895221 10.1097/RLI.0000000000000619 PMC7039314

[33] Navest RJ, Mandija S, Andreychenko A, Raaijmakers AJ, Lagendijk JJ, van den Berg CA. Understanding the physical relations governing the noise navigator. Magn Reson Med. 2019;82:2236–2247. 31317566 10.1002/mrm.27906 PMC6771522

[34] Speier P, Bacher M. Skip the Electrodes. But Not A Beat: The Engineering Behind the Beat Sensor Magnetom Flash; 2023: 106–113.

[35] Falcão MB, di Sopra L, Ma L, Pilot tone navigation for respiratory and cardiac motion-resolved free-running 5d flow mri. Magn Reson Med. 2022;87:718–732. 34611923 10.1002/mrm.29023 PMC8627452

[36] Ludwig J, Speier P, Seifert F, Schaeffter T, Kolbitsch C. Pilot tone–based motion correction for prospective respiratory compensated cardiac cine MRI. Magn Reson Med. 2020;85:1–14. 10.1002/mrm.28580 33226699

[37] Solomon E, Rigie DS, Vahle T, Free-breathing radial imaging using a pilot-tone radiofrequency transmitter for detection of respiratory motion. Magn Reson Med. 2021;85:2672–2685. 33306216 10.1002/mrm.28616 PMC7902348

[38] Wilkinson T, Godinez F, Brackenier Y, Motion estimation for brain imaging at ultra-high field using pilot-tone: comparison with DISORDER motion compensation. Proc Intl Soc Mag Reson Med. 2021;29:122.

[39] Maas SA. Nonlinear Microwave and RF Circuits. Artech House; 2003.

[40] Sporrer B, Wu L, Bettini L, A fully integrated dual-channel on-coil CMOS receiver for array coils in 1.5–10.5 T MRI. IEEE Trans Biomed Circuit Syst. 2017;11:1245–1255. 10.1109/TBCAS.2017.2764443 29293422

[41] Tang JA, Wiggins GC, Sodickson DK, Jerschow A. Cutoff-free traveling wave NMR. Concepts Magn Reson Pt A. 2011;38:253–267.

[42] Brunner DO, De Zanche N, Fröhlich J, Paska J, Pruessmann KP. Travelling-wave nuclear magnetic resonance. Nature. 2009;457:994–998. 19225521 10.1038/nature07752

[43] Seeber D, Jevtic J, Menon A. Floating shield current suppression trap. Concepts Magn Reson Pt B Magn Reson Eng. 2004;21B:26–31.

[44] Federal Communications Commission. CFR 2020 – title 47 – telecommunication. 2020 https://www.govinfo.gov/content/pkg/CFR-2020-title47-vol1/xml/CFR-2020-title47-vol1-sec1-1310.xml. Accessed February 12, 2024.

[45] Anand S, Lustig M. Wireless in-bore Ballistocardiography with 2.4GHz beat pilot tone (BPT). Proc Intl Soc Mag Reson Med. 2023;31:756.

[46] Marstal K, Berendsen F, Staring M, Klein S. Simpleelastix: a user-friendly, multi-lingual library for medical image registration. Paper presented at: International Workshop on Biomedical Image Registration (WBIR). 2016.

[47] Kellman P, McVeigh ER. Image reconstruction in SNR units: a general method for SNR measurement. Magn Reson Med. 2005;54:1439–1447. 16261576 10.1002/mrm.20713 PMC2570032

[48] Brablik J, Ladrova M, Vilimek D, A comparison of alternative approaches to MR cardiac triggering: a pilot study at 3 tesla. IEEE J Biomed Health Inform. 2022;26:2594–2605. 35085098 10.1109/JBHI.2022.3146707

[49] Noordegraaf A. Physical aspects of the “direct” recording of body displacement, velocity, and acceleration by shin-Bar Ballistocardiographs. Circulation. 1961;23:426–433. 13729490 10.1161/01.cir.23.3.426

[50] Nedoma J, Martinek R, Fajkus M, A novel FBG-based triggering system for cardiac MR imaging at 3 tesla: A pilot pre-clinical study. IEEE Access. 2020;8:181205–181223.

[51] Shao D, Tsow F, Liu C, Yang Y, Tao N. Simultaneous monitoring of ballistocardiogram and photoplethysmogram using a camera. IEEE Trans Biomed Eng. 2017;64:1003–1010. 27362754 10.1109/TBME.2016.2585109 PMC5523454

[52] Pinheiro E, Postolache O, Girão P. Theory and developments in an unobtrusive cardiovascular system representation: ballistocardiography. Open Biomed Eng J. 2010;4:201–216. 21673836 10.2174/1874120701004010201 PMC3111731

[53] March HW. Three-plane ballistocardiography: the effect of age on the longitudinal, lateral, and dorsoventral ballistocardiograms. Circulation. 1955;12:869–882. 13270345 10.1161/01.cir.12.5.869

[54] Blacher J, Asmar R, Djane S, London GM, Safar ME. Aortic pulse wave velocity as a marker of cardiovascular risk in hypertensive patients. Hypertension. 1999;33:1111–1117. 10334796 10.1161/01.hyp.33.5.1111

[55] Chen S, Sun H, Chen H, Anand S, Lustig M, Zhang Z. Towards contact-free motion sensing technique at low-field MRI using beat pilot tone. Proc Intl Soc Mag Reson Med. 2023;31:1020.

[56] Marques JP, Simonis FF, Webb AG. Low-field MRI: an MR physics perspective. J Magn Reson Imaging. 2019;49:1528–1542. 30637943 10.1002/jmri.26637 PMC6590434

[57] Roy CW, van Amerom JF, Marini D, Seed M, Macgowan CK. Fetal cardiac MRI: a review of technical advancements. Top Magn Reson Imaging. 2019;28:235–244. 31592990 10.1097/RMR.0000000000000218 PMC6791520

[58] Alon L, Dehkharghani S. A stroke detection and discrimination framework using broadband microwave scattering on stochastic models with deep learning. Sci Rep. 2021;11:24222. 34930921 10.1038/s41598-021-03043-y PMC8688451

