# Supplementary material for: Beat Pilot Tone (BPT): Simultaneous MRI and RF motion sensing at arbitrary frequencies
Source: Magn Reson Med. Author manuscript; Available in PMC 2025 Oct 1. (PMC11429784; doi:10.1002/mrm.30150)
Supplement: Supinfo — Figure S1. Intermodulation measurements on a preamp. The strength of the intermodulation product (IMD) was measured for two tones at 2.4 GHz (left) and 2.5278 GHz (right) on a preamplifier interface box for custom receiver coils (Clinical MR Solutions, LLC). Using a spectrum analyzer (FieldFox N9918A; Keysight Technologies), the input power was swept from −14 to +2dBm, and the power of the IMD was measured, along with the output power at 2.4 and 2.5278 GHz (“fundamental”). The second-order intercept point (IP2) where the lines cross was extrapolated based on fitting lines to the fundamental and IMD data. The measurements suggest that it is possible to obtain an IMD power close to that of the MR signal (>−70 dBm) with little BPT-Tx power (−10 dBm). Thus, intermodulation in the preamp is the likely mechanism of BPT-Rx, and there may not be significant gain suppression so far from the IP2 point. Figure S2. Bulk motion in the respiratory experiment. Bulk motion estimates from the respiratory experiment, with rotation angle on the left, and displacements on the right. The estimates were obtained by registering the images using SimpleElastix,46 a rigid transformation, and default rigid registration options (mutual information as the metric; multiresolution registration; stochastic gradient descent). The rotation was within ±3 degrees about the head-foot axis, and the displacement was between −15 and 5 mm in the left-right and anterior-posterior axes. Figure S3. BPT-Rx respiratory signal over frequency. A volunteer performed different breathing types (chest breathing, stomach breathing, rapid-shallow breathing, and breathing with simultaneous bulk motion of the chest). The experiment was repeated at BPT-Tx frequencies of 300/427.6, 800/927.6, 1200/1327.6, 1800/1927.6, and 2400/2527.6 MHz. (A) BPT-Rx signals with multiple peaks were manually chosen from each experiment, with coil positions in (B). There appears to be at least one coil signal with multiple peaks for each of [file NIHMS1993867-supplement-Supinfo.pdf]

Supporting Information for Beat Pilot Tone (BPT): Simultaneous MRI and RF motion sensing at arbitrary frequencies

1 BPT Phase Processing

The phase of the BPT data was wrapped due to scanner-specific phase variations such as B_0 eddy current compensation. We estimated the unwrapped phase by applying the pseudo-inverse of the relative phase operator $\mathbf{P}$ to the data. We derive the pseudoinverse for a single timepoint below; the same operations can be applied to all timepoints independently.

The BPT signal $\mathbf{X}$ at a given time point is an array of size $c \times 1$, where c is the number of receiver coils. Let $x_1, x_2, ..., x_c$ be the complex-valued BPT sample for each coil, respectively, at said time point. We can then write $\mathbf{X}$ as follows:

$$\mathbf{X} = \begin{bmatrix} x_1 \\ \vdots \\ x_c \end{bmatrix} \tag{1}$$

$$= \begin{bmatrix} a_1 e^{j\theta_1} \\ \vdots \\ a_c e^{j\theta_c} \end{bmatrix} \tag{2}$$

The goal is to recover the underlying unwrapped phase $\boldsymbol{\theta}$, where

$$\boldsymbol{\theta} = \begin{bmatrix} \theta_1 \\ \vdots \\ \theta_c \end{bmatrix} \tag{3}$$

Due to phase wrapping, $\boldsymbol{\theta} \neq \angle\mathbf{X}$. We can try to unwrap the data by applying an unwrapping operator $\mathbf{U}$ to $\angle\mathbf{X}$. $\mathbf{U}$ attempts to remove large discontinuities of more than π. However, this fails when there are many phase wraps. Instead, we can first compute the phase relative to a chosen

reference coil, then unwrap it. We compute the unwrapped relative phase vector $\mathbf{X_{ph,r}}$ as:

$$\begin{aligned}
\mathbf{X_{ph,r}} &= \mathbf{U}\angle\left[x_r^* \cdot \mathbf{X}\right] && (4)\\
&= \mathbf{U}\angle \begin{bmatrix} a_r e^{-j\theta_r} a_1 e^{j\theta_1} \\ \vdots \\ a_r e^{-j\theta_r} a_c e^{j\theta_c} \end{bmatrix} && (5)\\
&= \mathbf{U}\angle \begin{bmatrix} a_r a_1 e^{j(\theta_1-\theta_r)} \\ \vdots \\ a_r a_c e^{j(\theta_c-\theta_r)} \end{bmatrix} && (6)\\
&= \begin{bmatrix} \theta_1 - \theta_r \\ \vdots \\ \theta_c - \theta_r \end{bmatrix} && (7)\\
&= \boldsymbol{\theta} + \begin{bmatrix} 0 & -1 & \dots & 0 \\ \vdots & \vdots & \ddots & \\ 0 & -1 & \dots & 0 \end{bmatrix} \boldsymbol{\theta} && (8)\\
&= (\mathbf{I} + \mathbf{S_r})\boldsymbol{\theta}, && (9)
\end{aligned}$$

where $*$ denotes the complex conjugate and $\mathbf{S_r}$ consists of -1 in the rth column and 0s everywhere else. We can compute $\mathbf{X_{ph,r}}$ for all values of r and stack them into a matrix $\mathbf{X_{ph}}$:

$$\mathbf{X_{ph}} = \begin{bmatrix} \mathbf{X_{ph,1}} \\ \vdots \\ \mathbf{X_{ph,c}} \end{bmatrix} \qquad (10)$$

$$\begin{aligned}
&= \begin{bmatrix} \mathbf{I} + \mathbf{S_1} \\ \vdots \\ \mathbf{I} + \mathbf{S_c} \end{bmatrix} \boldsymbol{\theta} && (11)\\
&= \mathbf{P}\boldsymbol{\theta} && (12)
\end{aligned}$$

$\boldsymbol{\theta}$ can then be obtained by applying the pseudoinverse of $\mathbf{P}$ to $\mathbf{X_{ph}}$:

$$\boldsymbol{\theta} = \mathbf{P}^{\dagger}\mathbf{X_{ph}} \qquad (13)$$

This pseudoinverse operation was applied to the phase data in the respiratory experiment (Figure 6). Coil 10 was discarded from the data due to excessive wrapping.

2 Standing Wave Calculations

The maximum number of peaks in the standing wave is theoretically determined by the frequencies of the two fields and the length of the waveguide and serves as a validation of the simulation accuracy. In this section, we derive the expecting number of peaks in the standing wave based on

frequency. The standing wave patterns can be idealized as two sinusoids in space with amplitudes $\mathbf{A_1}$ and $\mathbf{A_2}$:

$$\mathbf{A_1} = a_1 cos(2\pi k_1 \mathbf{d} + \phi_1) \tag{14}$$
$$\mathbf{A_2} = a_2 cos(2\pi k_2 \mathbf{d} + \phi_2) \tag{15}$$

where a_1 and a_2 are constants, k_1 and k_2 are the wavenumbers corresponding to the two transmit frequencies, $\mathbf{d}$ is the longitudinal distance vector over the waveguide, and ϕ_1 and ϕ_2 are the phases for each sinusoid. The wavenumber or spatial frequency k_i is given by $2\pi f_i/c$, where c is the speed of light in air and f_i is the transmit frequency.
The amplitude of the BPT-Tx field over space can be modelled as the product of the two sinusoids:

$$\begin{aligned} \mathbf{A_{BPT}} =& \mathbf{A_1}\mathbf{A_2} \\ =& \frac{a_1 a_2}{2} \left[cos(2\pi(k_1 + k_2)\mathbf{d} + \phi_1 + \phi_2) \right. \\ & \left. + cos(2\pi(k_1 - k_2)\mathbf{d} + \phi_1 - \phi_2)\right] \end{aligned} \tag{16}$$

As Equation 16 suggests, the BPT-Tx amplitude can be written as the sum of a fast sinusoid with spatial frequency $k_1 + k_2$ in space and a slow sinusoid with spatial frequency $k_1 - k_2$. The fast sinusoid is amplitude-modulated by the slow sinusoid; therefore, the maximum number of peaks over a distance Δd is governed by the wavelength of the fast sinusoid, while the minimum number is governed by the slow sinusoid.
The wavelength of the fast and slow sinusoids are:

$$\lambda_{fast} = \frac{c}{f_1 + f_2} \tag{17}$$
$$\lambda_{slow} = \frac{c}{|f_1 - f_2|} \tag{18}$$
$$\tag{19}$$

Since there are two peaks in the magnitude over each sinusoid period,

$$\left\lfloor \frac{2\Delta d}{\lambda_{\text{slow}}} \right\rfloor \leq N_{\text{peaks}} \leq \left\lceil \frac{2\Delta d}{\lambda_{\text{fast}}} \right\rceil \tag{20}$$

where $\lfloor\rfloor$ and $\lceil\rceil$ denote the floor and ceiling operations, respectively. The slow sinusoid has the same frequency for all BPT-Tx pairs. If $f_2 - f_1 = 127.8$MHz, then the minimum number of peaks over a distance of 20cm is 0.

3 Vibration Artifact Removal

This section discusses an algorithm that may be used to remove artifacts caused by vibrations. For a single coil, the multi-frame BPT data $\mathbf{b}$ is of size $[N_f \times N_{pe}]$, where N_f is the number of frames and N_{pe} is the number of k-space lines. The artifact $\mathbf{l}$ can be modeled as:

$$\mathbf{l} = \alpha_0 \mathbf{c} + \alpha_1 \mathbf{y_{pe}} \tag{21}$$

where $\mathbf{y_{pe}}$ is a vector of phase encode indices which ranges linearly from -1 to 1 over a single frame and repeats over frames, and $\mathbf{c}$ is a constant-valued vector. The linear fit is approximated by solving a linear system of equations $\mathbf{A}\boldsymbol{\alpha} = \mathbf{b_f}$ for $\boldsymbol{\alpha}$, where:

$$\mathbf{A} = \begin{bmatrix} | & | \\ \mathbf{c} & \mathbf{y_{pe}} \\ | & | \end{bmatrix}, \tag{22}$$

$$\boldsymbol{\alpha} = \begin{bmatrix} \alpha_0 \\ \alpha_1 \end{bmatrix}, \tag{23}$$

and $\mathbf{b_f}$ is the flattened BPT data of size $[(N_{pe} \times N_f) \times 1]$. The corrected BPT signal is then $\mathbf{b_f} - \mathbf{l}$.

Figure 10c shows an example of one of the BPT signals in a 2-dimensional head motion experiment with and without artifact correction. Both uncorrected and corrected signals were low-pass filtered with a cutoff of 5Hz. For the respiratory and head data in this paper, low-pass filtering and averaging, respectively, were sufficient to correct for the artifact.

Table S1: Type and placement of BPT and PT antennas

Experiment	BPT Type	PT Type	BPT Placement	PT Placement
Rocker	Log periodic	–	Outside	–
Respiratory	4G LTE	Loop	Top of bore	Top of bore
GEM Cardiac	PCB	Loop	Top of bore	On chest
dBCG	PCB	Loop	Top of bore	On chest
AIR Cardiac	4G LTE	Loop	On chest	–
Head	2 × PCB	–	Top and side of coil	–

This table shows the type and placement of antennas for BPT and PT. Double dashes (–) indicate that the antenna or placement was the same, e.g., the same log periodic antenna was used to transmit both BPT and PT.

Table S2: 2.4GHz BPT Hardware

Hardware element	Model	Company
Transmitter	USRP B200	Ettus Research
Power combiner	ZN2PD-63+	Minicircuits
Amplifier	Sunhans 2.4GHz 34dBm WiFi Booster	Sunhans
High-pass filter	VHP-19	Minicircuits

Table S3: PC correlation values

Reg-PC	BPT-Top	BPT-Side	BPT-Both	PT-Top	PT-Side	PT-Both
0	0.952	0.970	**0.991**	0.930	0.959	0.984
1	0.842	0.920	**0.973**	0.730	0.728	0.970
2	0.968	0.957	**0.992**	0.943	0.954	0.990

We regressed the multicoil BPT-Rx and PT signals to each reg-PC using data from the top antenna alone, the side antenna alone, and both antennas. We report the Pearson correlation coefficient of each reg-PC with each regressed BPT-Rx/PT. Bolded values indicate the maximum correlation value with each reg-PC.

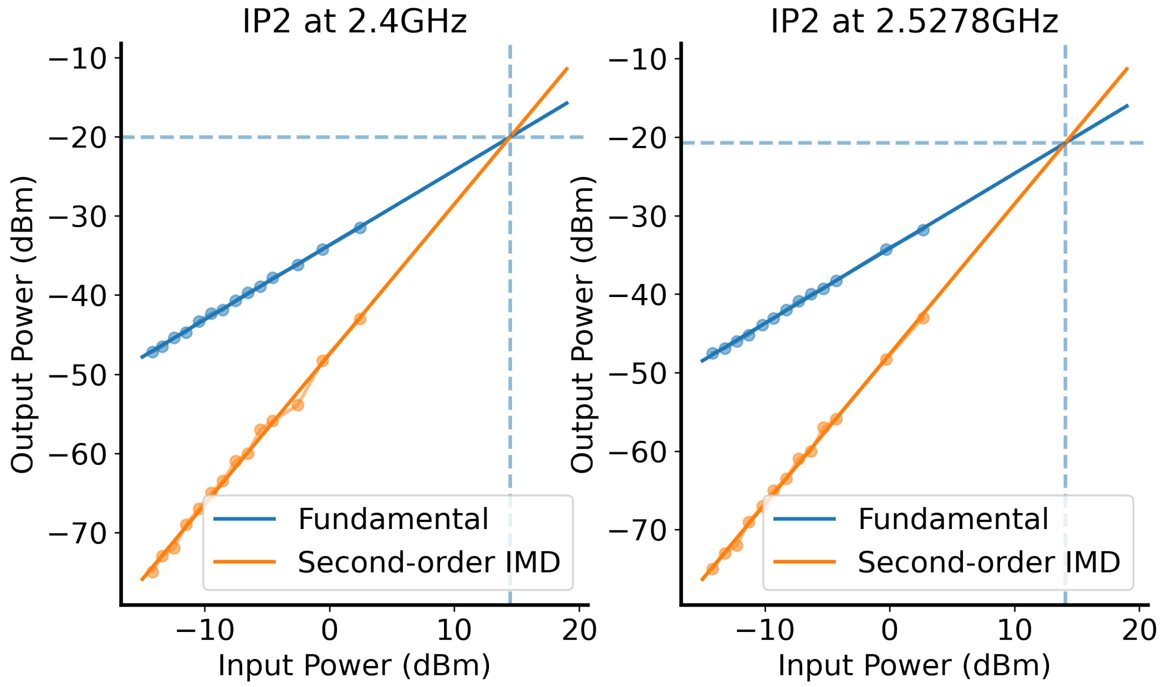

Figure S1: **Intermodulation measurements on a preamp**. The strength of the intermodulation product (IMD) was measured for two tones at 2.4GHz (left) and 2.5278GHz (right) on a preamplifier interface box for custom receiver coils (Clinical MR Solutions, LLC). Using a spectrum analyzer (FieldFox N9918A; Keysight Technologies, the input power was swept from -14dBm to +2dBm, and the power of the IMD was measured, along with the output power at 2.4 and 2.5278GHz ("fundamental"). The second-order intercept point (IP2) where the lines cross was extrapolated based on fitting lines to the fundamental and IMD data. The measurements suggest that it is possible to obtain an IMD power close to that of the MR signal (> -70dBm) with little BPT-Tx power (-10dBm). Thus, intermodulation in the preamp is the likely mechanism of BPT-Rx, and there may not be significant gain suppression so far from the IP2 point.

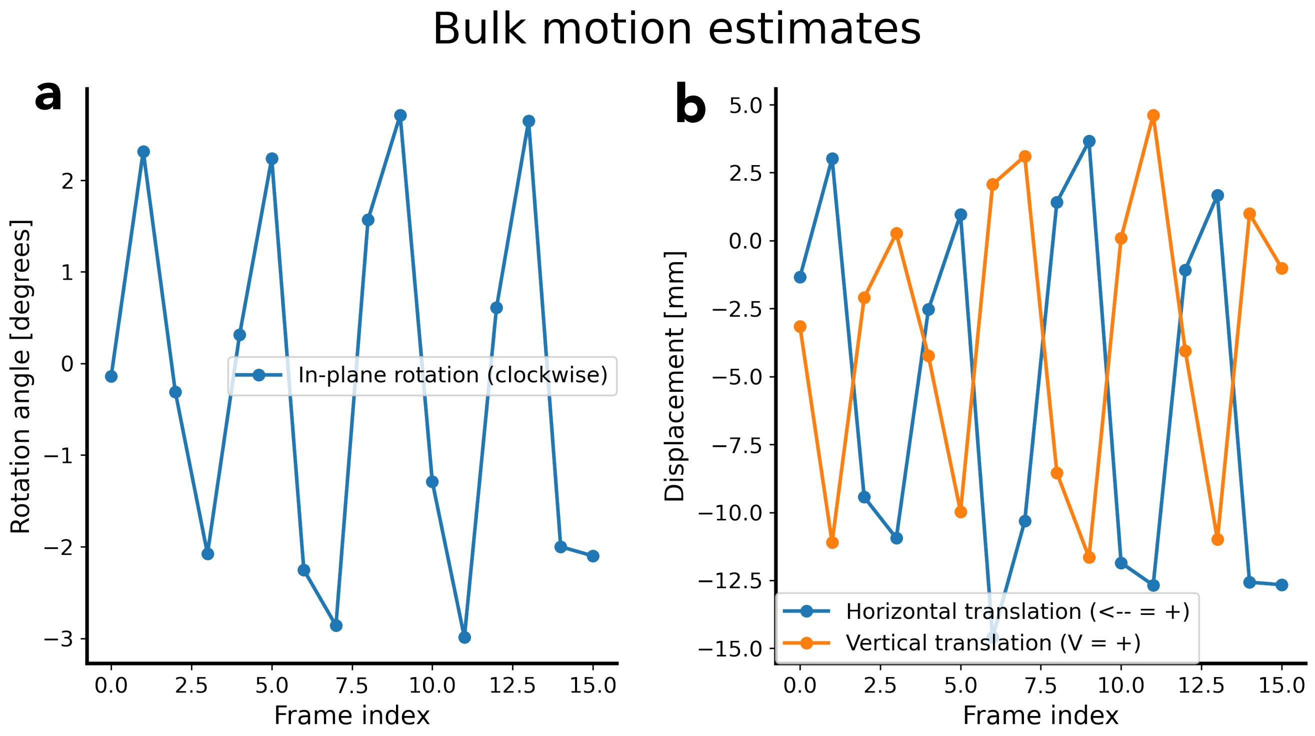

Figure S2: **Bulk motion in the respiratory experiment.** Bulk motion estimates from the respiratory experiment, with rotation angle on the left, and displacements on the right. The estimates were obtained by registering the images using SimpleElastix, a rigid transformation, and default rigid registration options (mutual information as the metric; multi-resolution registration; stochastic gradient descent). The rotation was within +/- 3 degrees about the head-foot axis, and the displacement was between -15 and 5 mm in the left-right and anterior-posterior axes.

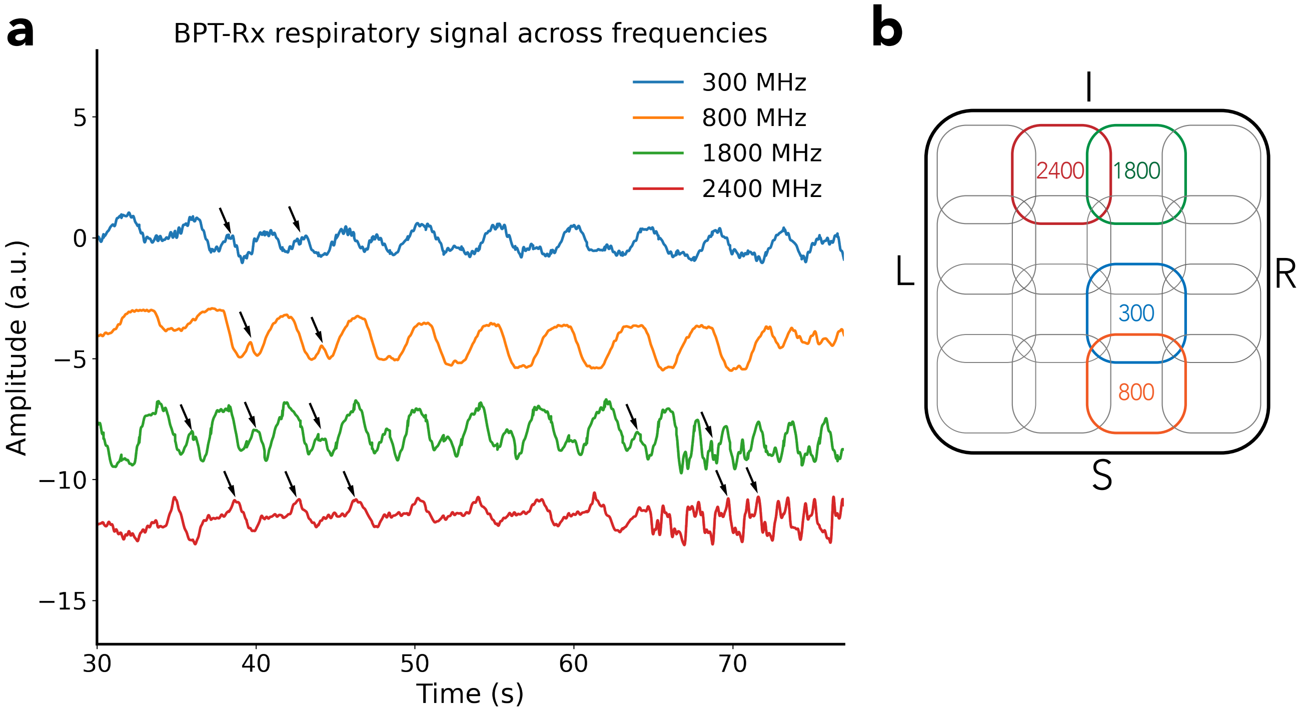

Figure S3: **BPT-Rx respiratory signal over frequency**. A volunteer performed different breathing types (chest breathing, stomach breathing, rapid-shallow breathing, and breathing with simultaneous bulk motion of the chest). The experiment was repeated at BPT-Tx frequencies of 300/427.6MHz, 800/927.6MHz, 1200/1327.6MHz, 1800/1927.6MHz, and 2400/2527.6MHz. a) BPT-Rx signals with multiple peaks were manually chosen from each experiment, with coil positions in b). There appears to be at least one coil signal with multiple peaks for each of the frequencies, except for 1200/1327.6MHz. These peaks could be due to the shape of the BPT-Tx standing wave pattern.

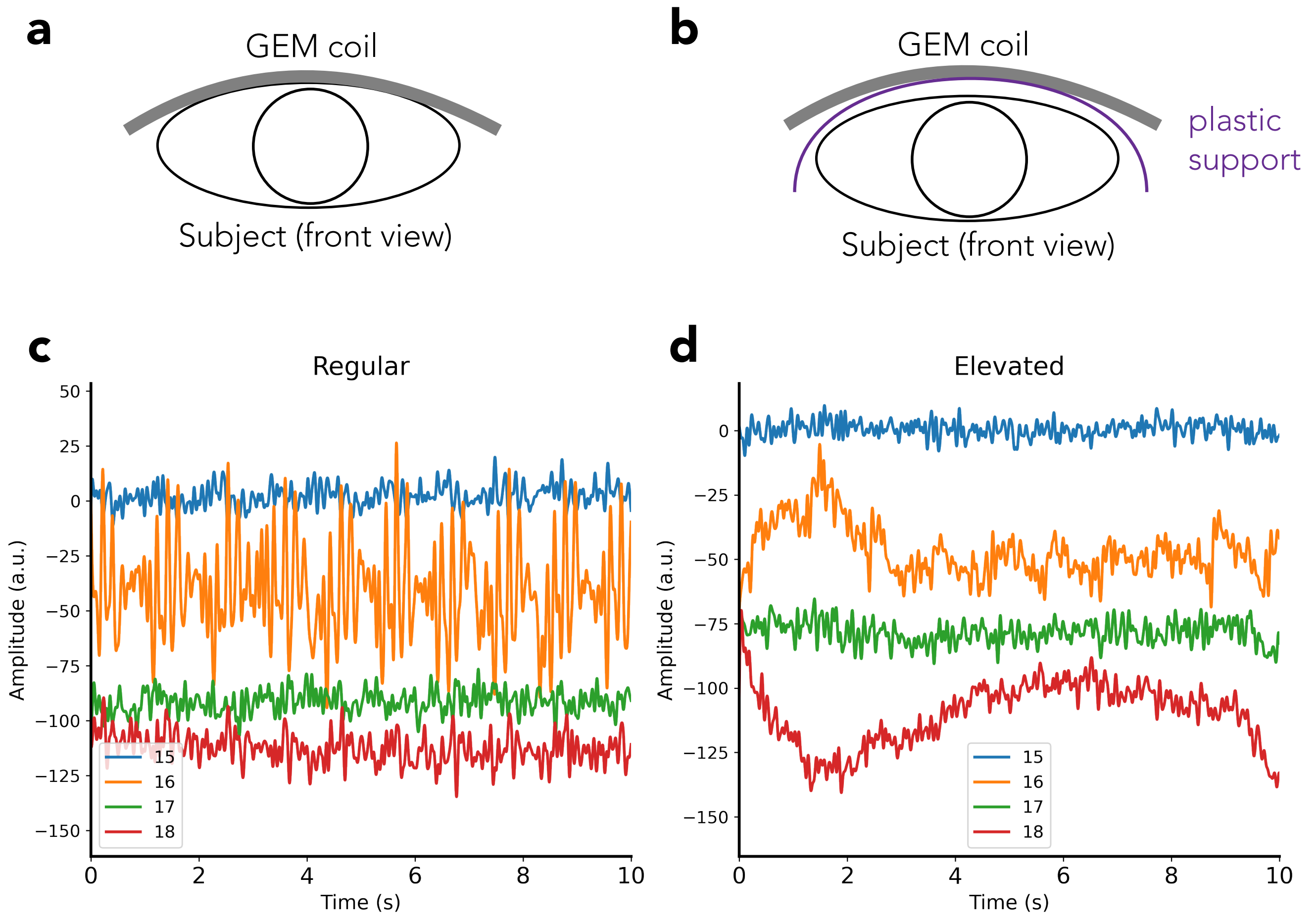

Figure S4: **Rigidly contacting vs elevated GEM coil cardiac experiment**. Breath-held cardiac BPT-Rx signals were acquired on a volunteer with a) the GEM anterior array (AA) coil placed on the chest and b) elevated from the chest using a plastic support with BPT-Tx at 2.4/2.5278 GHz. BPT signals from selected AA coils in c) the normal configuration and d) the elevated configuration. The elevated signals show lower cardiac modulation, and perhaps no longer a dBCG signal.

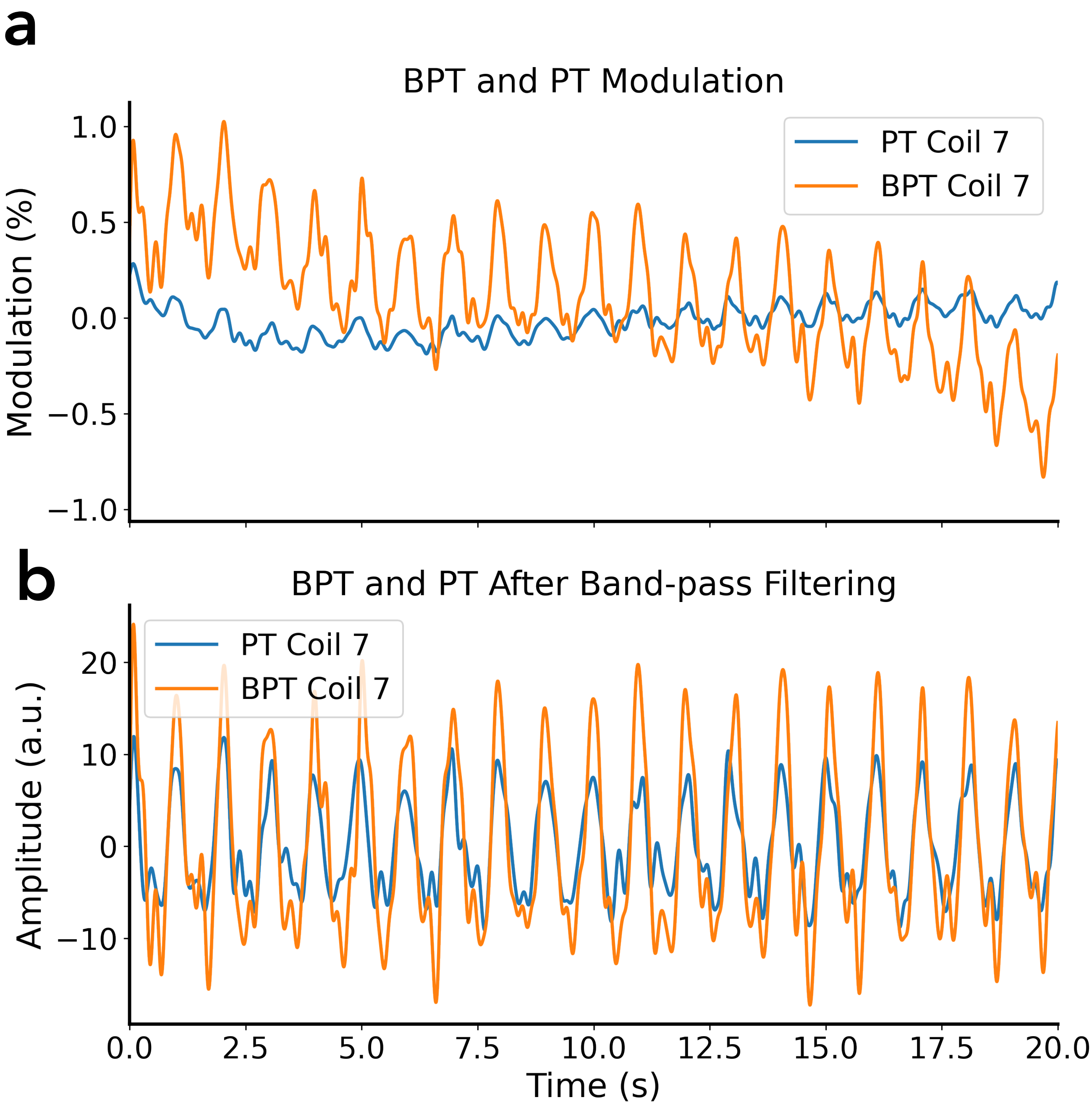

Figure S5: **AIR coil results**. BPT-Rx and PT were acquired simultaneously in a breath-held scan with RF excitation and gradients off using an abdominal AIR coil. The BPT-Tx frequencies were 1.8/1.9298GHz, and PT was 129.6MHz. Top: BPT-Rx and PT are plotted for a single coil in percent modulation units after being low-pass filtered with a cutoff of 5Hz. Bottom: The same BPT-Rx and PT data after band-pass filtering between 0.5 and 5Hz. Both appear comparable in terms of signal shape and modulation level.

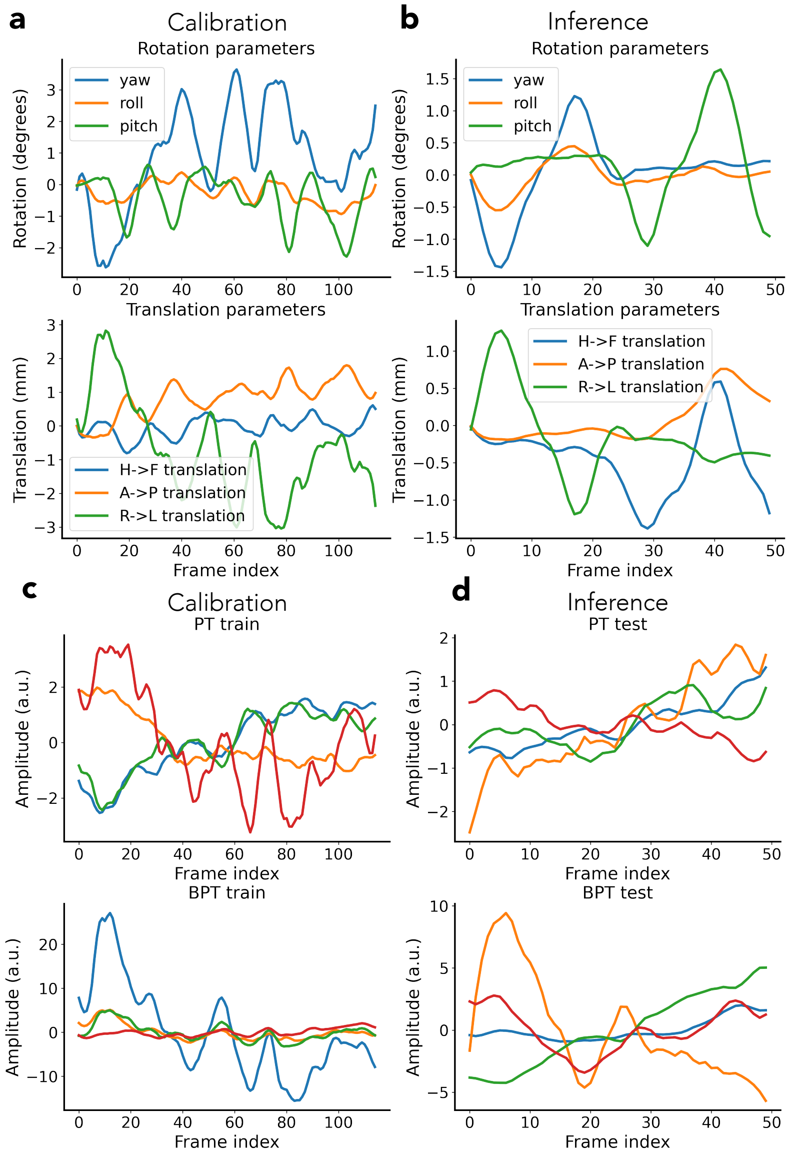

Figure S6: **Motion parameters during the MIMO head motion experiment.** In the calibration scan, the subject attempted to capture the full rigid parameter space (i.e., all possible translations and rotations within an approximate range of +/-3mm and +/- 4 degrees) by moving in a raster fashion. In the inference scan, they shook their head "no" and nodded "yes". PT and BPT-Rx data from both antennas were combined across antennas and averaged over frames. Rigid motion parameters were estimated from registering the images from a) the calibration scan and b) the inference scan after Savitzky-Golay filtering (window size = 11, order = 4). PT (top) and BPT (bottom) from the four most modulated coils are plotted from the c) the calibration scan and d) the inference scan after Savitzky-Golay filtering (window size = 11, order = 4).

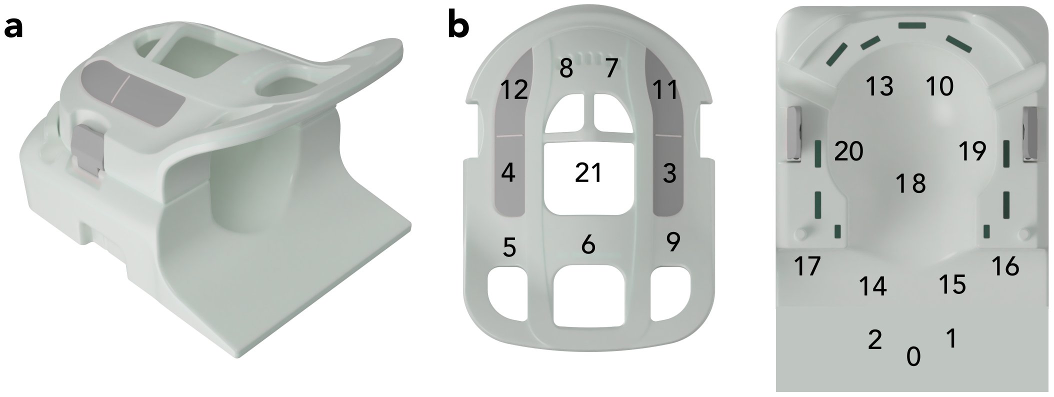

Figure S7: **GEM head coil arrangement**. a) Rendering of the GEM Head and Neck Unit (HNU) coil used for the head motion experiment. b) Approximate physical arrangement of the 22 coil elements. Elements 0, 1, and 2 belong to the posterior array integrated into the scanner bed.

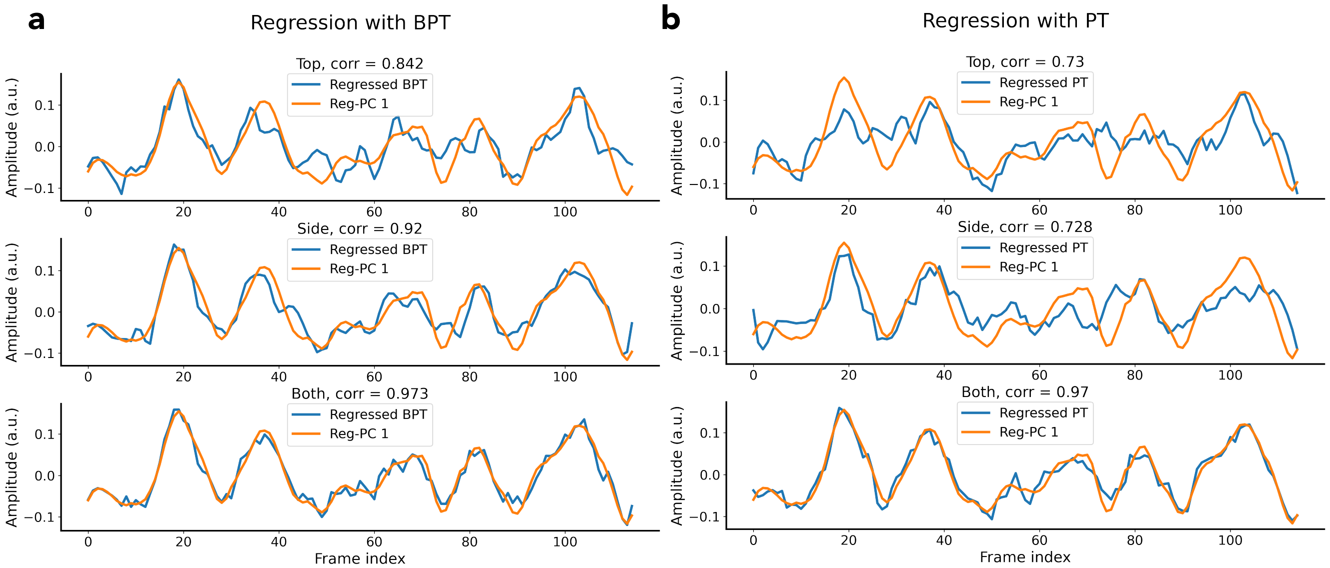

Figure S8: **BPT-Rx and PT regression to reg-PC 1.** Filtered and concatenated data were regressed to reg-PC 1 for a) BPT-Rx and b) PT data. The top row is for data from the top antenna, the second row for the side antenna, and the bottom row for both. Pearson correlation coefficient is reported for each subplot. The correlation is higher for PT and BPT-Rx when using data from both antennas and is higher for BPT-Rx than for PT.

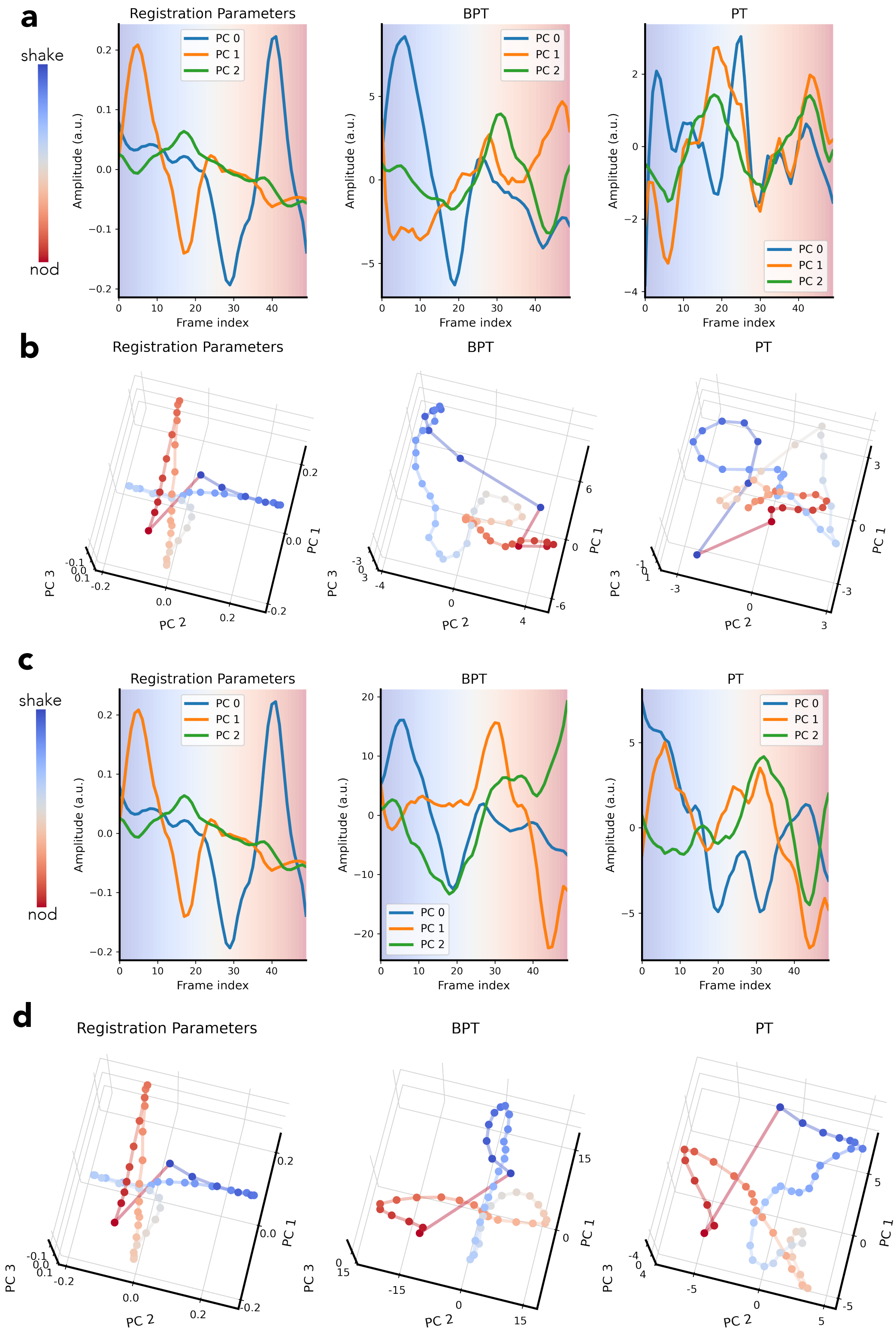

Figure S9: **PCs with different antennas vs combined.** a) BPT and PT PCs computed only from the antenna at the top. The PCs from registration were unchanged. b) BPT and PT PCs from the top antenna in the PC space. c) BPT and PT PCs computed from the side antenna vs time and d) in the PC space.
